# An exploration of error-driven learning in simple two-layer networks from a discriminative learning perspective

**DOI:** 10.3758/s13428-021-01711-5

**Published:** 2022-01-14

**Authors:** Dorothée B. Hoppe, Petra Hendriks, Michael Ramscar, Jacolien van Rij

**Affiliations:** 1 Center for Language and Cognition, University of Groningen, Groningen, The Netherlands; 2 Department of Linguistics, University of Tübingen, Tübingen, Germany; 3 Department of Artificial Intelligence, University of Groningen, Groningen, The Netherlands

**Keywords:** Error-driven learning, Discriminative learning, Computational simulations, Cognitive modeling, Neural network models

## Abstract

Error-driven learning algorithms, which iteratively adjust expectations based on prediction error, are the basis for a vast array of computational models in the brain and cognitive sciences that often differ widely in their precise form and application: they range from simple models in psychology and cybernetics to current complex deep learning models dominating discussions in machine learning and artificial intelligence. However, despite the ubiquity of this mechanism, detailed analyses of its basic workings uninfluenced by existing theories or specific research goals are rare in the literature. To address this, we present an exposition of error-driven learning – focusing on its simplest form for clarity – and relate this to the historical development of error-driven learning models in the cognitive sciences. Although historically error-driven models have been thought of as associative, such that learning is thought to combine preexisting elemental representations, our analysis will highlight the discriminative nature of learning in these models and the implications of this for the way how learning is conceptualized. We complement our theoretical introduction to error-driven learning with a practical guide to the application of simple error-driven learning models in which we discuss a number of example simulations, that are also presented in detail in an accompanying tutorial.

## Introduction

Error-driven learning models have been widely used in the fields of animal and human learning for several decades (see, e.g., Carpenter & Grossberg, 1987; McClelland & Rumelhart, 1981; Pearce & Hall, 1980; Rumelhart et al., 1986; Rescorla & Wagner, 1972; Rosenblatt, 1962; Rumelhart, Hinton, & Williams, 1986; Sutton & Barto, 1998; Widrow & Hoff, 1960). They have also become the dominant approach in machine learning research, with error-driven learning mechanisms forming the core of today’s most popular AI applications based on artificial neural networks (such as, e.g., OCR reading, LeCun, Bottou, Bengio, & Haffner, 1998; machine translation, Wu et al., 2016; or autonomous driving, Pomerleau, 1988). However, given the high complexity of most of these latter models, theoretical discussions of applications of error-driven learning mostly focus on the further optimization of network architectures, while the core learning mechanisms receive little attention, being largely taken for granted. As a result, despite their omnipresence, error-driven learning mechanisms are rarely the subject of theoretical investigation in the domains in which they are applied.

Given the fundamental role of error-driven mechanisms in cognitive science, and, as we describe below, the somewhat haphazard way in which this role has emerged, we suggest that there is much to be gained from taking a step back and revisiting the core mechanism, its workings and their relation to theories of cognition in more detail. Although an understanding of the basic error-driven mechanism should be a critical prerequisite to the application and generation of learning models on any level of complexity, it is clear (see Ramscar, Yarlett, Dye, Denny, & Thorpe, 2010) that many misapprehensions about error-driven learning — for example, that it is a form of associative learning (as it is conceptualized by, e.g., Rescorla & Wagner, 1972) — persist in the literature.

Note that model simplification is perhaps easily stated as a general solution; it is still important to acknowledge that while error-driven learning mechanisms are deceptively simple, models based on these mechanisms only tend to be useful in the context of complex architectures. This is an old theoretical issue. While complex models often provide a higher degree of performance, interpretability is usually lost with increasing model complexity (*Bonini’s paradox*; Dutton & Starbuck, 1971). Accordingly, much of our focus here will be on the basics of the simple mechanism at the heart of these models, rather than on complex architectures.

A further issue complicating the understanding of error-driven learning mechanisms is that information about them tends to be scattered over the literature of different fields and appears in many different theoretical contexts. The fact that error-driven learning models have been integrated in many different theories makes it especially difficult to differentiate between theory- or application-specific parts of model specifications and parts that are in fact essential to the basic error-driven learning mechanism (which is a known problem in modeling, Cooper & Guest, 2014; McCloskey, 1991). Proposed error-driven models differ widely, not only in their network architecture, but also in their specific implementation of the learning mechanism, in the way model responses are interpreted and, last but foremost, in how they define a learning problem in terms of the input and output representations given to the model. Accordingly, we will describe how understandings of basic error-driven learning have been diluted by varying specifications in countless applications, which has often led to the potential of simple architectures being ignored. Yet, as we will show, many recent investigations have been successful in generating theoretical predictions and explanations employing only the simplest error-driven learning architectures, underlining the importance of careful theoretical analysis in this domain.

In what follows, we present a critical theoretical review of the basic error-driven learning mechanism and its relation to human cognition. Our aim will not be to offer an extensive literature review, but rather to present a theoretical characterization of the core error-driven learning mechanism, based on which we will then provide an overview of the scope of this learning mechanism. To this end, we will seek to contrast historical use and interpretation of simple error-driven learning models with recent advances, highlighting the way that current explorations of the basic dynamics of the learning mechanism have informed new theoretical insights about learning.

An important point that we will emphasize in presenting this model is that it — and all error-driven learning models that enforce cue and outcome competition (discussed in more detail in Sections “?? ??” and “Learning dynamics”) — belong to the class of *discriminative models* and implement a *discriminative learning mechanism*. As a preliminary, it will be important to clarify some historical ambiguities regarding the definition of *discriminative learning*.

- The term *discrimination learning* was initially used in the literature on animal learning and behavior (refer to, e.g., Hilgard & Bower, 1975). Consistent with the behaviorist principles that dominated theory in the earlier parts of the twentieth century, it was used in an externally grounded, mechanism-neutral way, to describe the requirement for animals and humans to be able to learn different responses to different stimuli.
- Later, in machine learning, the notion of a *discriminative model* was introduced to provide a more mathematical and more concrete conceptualization of discrimination learning in relation to classification problems. Unlike generative models — which they are contrasted with — discriminative models are simply defined in terms of their capacity to learn to maximize the conditional probabilities of output units given input units (Ng & Jordan, 2002). Importantly, this definition is once again neutral with regards to the mechanism, and while most classification problems in which discriminative models are employed also tend to implement discriminative algorithms (discussed below) this does not need to be the case (in fact, discriminative models are also sometimes referred to as *conditional models* as a reflection of this as, for example, in Gudivada, 2018)
- Finally, the mechanism which in most cases is implemented in discriminative models is some kind of *discriminative learning algorithm*, such as the error-driven learning algorithm we will analyze in this article. In most learning situations these kinds of learning mechanisms enforce cue and outcome competition, which together serve to discriminate against or in favor of the units that serve as input representations — by re-weighting the influence of individual units — which effectively rerepresents them according to how informative they are about different outputs (Ramscar et al., 2010).

Note, however, that it follows from all this that a discriminative learning algorithm is not always necessary to explain discrimination learning phenomena or to solve the classification problem stated by a discriminative model. As we will seek to elaborate in the course of this article, these points are highly dependent on the task and task structure in question. Although these different notions of discriminative learning have crucial implications for our conceptualization of the learning process, historically they have been obscured or ignored when models of “discrimination learning” have been applied to behavior, leading to a number of confusions about the strengths and weaknesses of the discriminative learning algorithms actually implemented in error-driven learning models.

Importantly, while historical treatments of learning have often employed error-driven learning mechanisms, at a theoretical level these treatments (e.g., Rescorla & Wagner, 1972) have still tended to be framed from an associative perspective guided by compositional principles, such that representations given to these models have been assumed to be combinations of preexisting low-level input representations (i.e., features, elements, or even microfeatures). However, as we discuss in detail below, this directly contradicts the logic of learning in error-driven learning models which indicates that representations depend on the learning process, which is guided by principles of discrimination rather than compositionality. Accordingly, one goal of this article will be to clarify this point, not only when examining the learning mechanism but especially when thinking about the nature of input representations and how the representations given to a model affect what this model can learn (Bröker & Ramscar, 2020).

In order to perform this theoretical review, it will be necessary to thoroughly discuss all of the important methodological components of simple error-driven learning models. Accordingly, as well as providing a theoretical introduction to the topic, this article also serves as a practical introduction to the method of modeling with minimal error-driven learning networks, especially together with the practical tutorial (https://dorohoppe.github.io/tutorials/edl.html) that we have compiled to accompany the article. Our aim is thus not just restricted to giving an idea of the scope of error-driven learning; we also hope to suggest ways to further explore the many possibilities offered by this mechanism.

### A brief history of error-driven learning

We will begin by briefly reviewing the development and subsequent use of error-driven learning in the fields of cognitive modeling and machine learning over the last 60 years.

Error-driven learning mechanisms were first introduced into cognitive science in order to provide a formalism which could account for the findings of early experiments in classical conditioning (e.g., Pavlov, 1927; Kamin, 1969; Rescorla, 1968) and discrimination learning (e.g. Krechevsky, 1932). In particular, one basic principle informed by experimentation changed the former understanding of associative learning and built the base of the theory behind error-driven learning: learning depends on how well a stimulus *predicts* a following response or a subsequent stimulus and not on mere temporal contingency (Rescorla, 1988). This principle followed directly from the observation that during learning not only stimuli that occur together are *associated* but also stimuli that do not occur together are *dissociated* (Rescorla, 1968). Subsequently developed models implemented these learning dynamics using a simple feed-forward two-layer^1^ artificial neural network in which weights between an input and output layer were updated with a learning mechanism that minimizes prediction error.

One of the first formulations of this error-minimization technique that gained widespread attention was the so-called “delta rule” by Widrow and Hoff (1960). A different formulation of this idea was subsequently presented by Rosenblatt (1962) integrated in one of his perceptron models (*γ*-perceptron). A decade later, Rescorla and Wagner (1972) published their model which basically implements a version of Widrow and Hoff’s (1960) delta rule with some additional assumptions. While these three simple models differed in some assumptions and parameters, they all employed two-layer feed-forward networks and an error-minimizing learning rule.

Soon, the field of interest of these early models outgrew animal learning (Miller, Barnet, & Grahame, 1995) and error-driven learning models were used to investigate human cognition (Gluck & Bower, 1988; Rumelhart & McClelland, 1986). From there, the models had been extended in many directions, among others: the addition of hidden layers and recurrent connections led to modern artificial neural networks with a generalized delta rule — backpropagation (McLaren, 1993; Rumelhart et al., 1986); representations of input and output units developed from elemental (low-level perceptions; McLaren & Mackintosh, 2000; Rescorla & Wagner, 1972; Wagner & Brandon, 2001) to configural (combinations of elemental features; Pearce, 1987; 2002); attention modulation mechanisms were added to account for more learning phenomena, such as latent inhibition (Harris, 2006; Mackintosh, 1975; Pearce & Hall, 1980); furthermore, temporal generalization (Sutton & Barto, 1987) led to the development of reinforcement learning (Sutton & Barto, 1998).

Early on the simple two-layer error-driven learning networks were harshly criticized for being too restricted and limited in scope, starting with a review of Rossenblatt’s (1962) basic perceptron by Minsky and Papert (1969). Especially regarding practical applications, multi-layer networks turned out to be much more powerful and successful with their ability to learn non-linear structure in the input by constructing intermediate abstract representations. The further exploration of the scope of the basic underlying error-driven learning rule bare of any extensions or modifications was, therefore, put on hold.

While research on the fundamentals of error-driven learning has never completely stopped, recent advances have revisited the original, simple models, in particular the Rescorla-Wagner model (Rescorla & Wagner, 1972), and questioned the theory and assumptions behind them. This has led to a number of new insights about fundamental properties of learning in developmental psychology (Ramscar, Dye, Gustafson, & Klein, 2013; Ramscar, Dye, & Klein, 2013; Ramscar, Thorpe, & Denny, 2007; Ramscar et al., 2010), aging research (Ramscar, Hendrix, Shaoul, Milin, & Baayen, 2014; Ramscar, Sun, Hendrix, & Baayen, 2017) and linguistics (Arnold, Tomaschek, Sering, Lopez, & Baayen, 2017; Arnon & Ramscar, 2012; Baayen, Milin, Ðurđević, Hendrix, & Marelli, 2011; Baayen, Shaoul, Willits, & Ramscar, 2016b; Milin, Divjak, & Baayen, 2017; Linke, Bröker, Ramscar, & Baayen, 2017; Ramscar, Dye, & McCauley, 2013; Ramscar & Dye, 2009; Ramscar & Yarlett, 2007; Nixon, 2020; Nixon & Tomaschek, 2020; St Clair, Monaghan, & Ramscar, 2009). Surprisingly, many of these simple models turn out to be able to explain seemingly complex phenomena of human cognition and sometimes even predict behavior that more optimal and rational models or more complex networks fail to explain (Gluck & Bower, 1988; Gureckis & Love, 2010). Hence, while error-driven learning is used in cognitive modeling and machine learning since over 60 years and integrated in highly complex models, the current findings with minimal error-driven models suggest two things: first, that the scope of the basic error-driven learning mechanism has still not been sufficiently explored; and second, that such a fundamental exploration is best done with radically simplified models. One likely reason for the former is, as we noted above, the widespread misconception that error-driven learning is associative (see, e.g., Harris, 2006; Miller et al., 1995; Rescorla & Wagner, 1972 ), and a further advantage of the latter is that the discriminative logic of error-driven learning can be illustrated most clearly in a simple model.

### Focus on the core learning mechanism

In order to make the workings of simple models more straightforward and easy to understand, in this article we will present an error-driven learning model that is stripped off all unnecessary assumptions and parameters (and layers).

In complex modern artificial neural network models it is becoming increasingly difficult to pinpoint which part of a model contributes to its behavior, and interestingly, there is currently a trend towards developing methods for analyzing the workings of complex neural networks (e.g., Adi, Kermany, Belinkov, Lavi, & Goldberg, 2016; Lei, Barzilay, & Jaakkola, 2016). The present approach is consistent with this in that our focus will be on the very simplest form of error-driven learning that nevertheless lies in the heart of these more complex systems.

That previous approaches of studying error-driven learning in two-layer network models haven’t advanced much over the last decades might have been partly due to scarce computational resources in the last century and partly due to the sociological dynamics surrounding the debate about the limitations of simple error-driven learning models (Olazaran, 1996). However, we will argue here that, to a large part, this was also caused by the too restricted specification of models, especially of the prominent Rescorla-Wagner model. On the one hand, we will show how the Rescorla-Wagner learning rule can be further simplified (see Section “?? ??”). On the other, we will show how the specific assumptions about the input and output representations used, strongly limited the scope of previous models (see Section “Cue and outcome representations”).

The aims of this article are to systematically introduce a simplified modeling framework to study error-driven learning and to summarize the resulting learning dynamics including their implications for learning theory. Crucially, this analysis of simple error-driven learning models will emphasize the discriminative logic of learning in them which, as we will highlight in the following, differs considerably from traditional conceptualizations of these models. In the course of this we thus seek to highlight the connections and, more importantly, the differences between this analysis and previous research with simple error-driven learning models.

We will start with a derivation of a simplified network architecture and learning rule which can effectively isolate the error-driven learning mechanism. Then, we will present the main learning dynamics arising from this setup and discuss how simulation results can be related to data of real learners to inform theories about learning. Finally, we will discuss the important role of input and output representations for the scope of the present discriminative error-driven learning model compared to historical models.

For readers interested in the practical implementation of cognitive modeling with minimal two-layer error-driven networks, we have prepared a tutorial which complements the theoretical aspects of the paper and provides the code and practical details about the employed examples (https://dorohoppe.github.io/tutorials/edl.html; the source code to the tutorial can be found at https://git.lwp.rug.nl/p251653/error-driven-learning-tutorial). An implementation of the present approach is available with the R package edl (van Rij & Hoppe, 2021).

## Network architecture and learning mechanism

The simple form of error-driven learning we will present here is a fully connected two-layer feed-forward neural network with a linear identity activation function and an incremental error-driven updating of connection weights, most widely known as the delta rule (McClelland, Rumelhart, & the PDP Research Group, 1986; Widrow & Hoff, 1960).

In the course of presenting this basic form of error-driven learning, we will also analyze its conceptual logic, and highlight the similarities and differences between our analysis and previous, historical work employing this mechanism. Before we begin, however, there is one important point we need to foreshadow: As we will see later (e.g., in Section “Cue and outcome representations”), the definition of the network architecture and learning mechanism is not enough to specify an error-driven learning model. The crucial part that sets the new discriminative perspective on these models off from previous perspectives is the treatment and interpretation of the input representations these models operate on.

### Error-driven learning is discriminative

Error-driven learning implements the idea that learning is based on expectations and is basically a process of making and testing predictions. Ultimately its aim is to reduce uncertainty about upcoming states of the world, which is also the objective of the larger class of discriminative models. In the following we will elaborate on the basic components of error-driven learning models that give rise to a mechanism satisfying this aim.

**Fig. 1:**
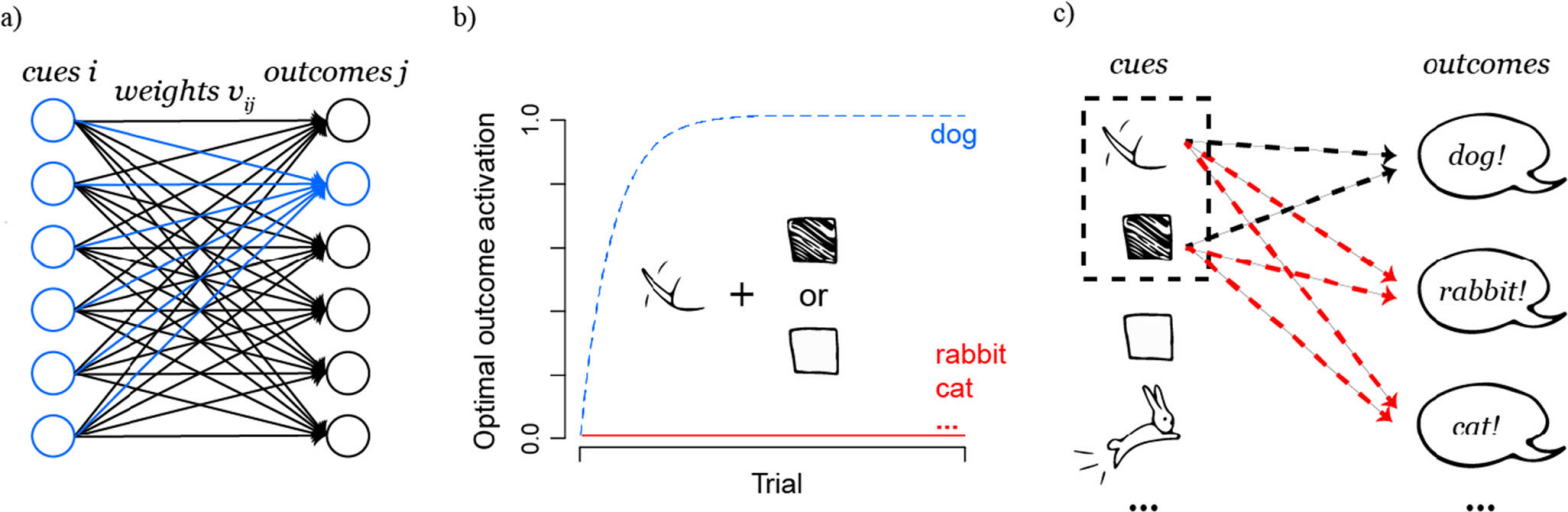
A fully connected error-driven learning network, with incoming connections to one outcome highlighted in blue (a). Consider an example of learning to discriminate animals by first seeing an animal, for example a dog, and then hearing it’s species name. (b) shows how the activation of the outcomes *dog* and other animals develops given the cue set *{tail-wagging, a specific fur color}*, maximizing certainty to expect one specific outcome. (c) shows a hypothetical weight update after seeing a dog and hearing “dog”. Black dashed lines show positive weight adjustments and red dashed lines negative adjustments. The dashed box shows the current cue set in which weights compete with each other

First of all, just as predictions, error-driven learning is directional and crucially, its unidirectional dynamics can only arise in a feed-forward network, in which connections are directed from input to output units. Hence, the basic model architecture we discuss in this paper is a feed-forward two-layer network that fully connects a layer of discrete input units (*cues*) with a layer of discrete output units (*outcomes*)^2^ as illustrated in Fig. 1a. As defined by the feed-forward property, weights from cues to outcomes are always directional, *from* a cue *to* an outcome, never bidirectional, *between* a cue and an outcome. This is the most basic architecture of a neural network, employed by most early error-driven learning models, for example, Widrow and Hoff’s (1960) ADALINE, Rosenblatt’s (1962) simple perceptron, and Rescorla and Wagner’s (1972) model.

Furthermore, as opposed to generative models which estimate a probability distribution over all previous data points, discriminative models update expectations primarily based on the most recent data point, while previous experience is captured only indirectly by the current state of the network. A key feature of error-driven learning is therefore also that it is an incremental algorithm that updates weights *online*: over discrete training trials, weights are incrementally updated recording a weight matrix for every point in time. The weight matrix *V* between cues *i* and outcomes *j* at time *t* is updated by adding a weight adjustment to yield the new state of the network at time *t* + 1:

1 $$  V_{ij}^{t+1}=V_{ij}^{t}+{{\varDelta}} V_{ij}^{t} $$

To motivate how the weight adjustment ${{\varDelta }} V_{ij}^{t}$ is calculated, we need to consider the goal of error-driven learning, which ultimately makes it discriminative, in more detail: to *reduce uncertainty* about the occurrence of states in the world (Gallistel, 2003; Ramscar, 2013; Rescorla, 1988). This means that ideally, at any point in time, the current state of the world will be expected with full certainty, given the current context or conditions. In order to reach this goal, a learner needs to learn to *discriminate* structures in the world according to how they predict *different* states of the world. For example, with what we have learned by seeing many different dogs and rabbits we are normally able to tell the difference between a dog and a rabbit with full certainty, taking into consideration their appearance and behavior. In terms of an error-driven learning system this means that ideally weights develop such that every possible set of cues, which can for example represent the features of an instance of a specific dog, fully predicts one unique outcome or outcome set, for example the word “dog” (see Fig. 1b).

Error-driven learning tries to achieve optimal discrimination of cue structures by minimizing the error between the desired state of full certainty about an outcome and the actual current expectation of this outcome to occur given the cues that are present at that point in time.

The desired full expectation of an outcome or the target value of the optimization process is usually formalized as the maximal activation (here, 1) of a specific outcome unit, and the minimal activation (here, 0) for not expected outcome units, respectively.

The actual current expectation of an outcome is captured by the *activation* of the outcome given the currently present cues. The activation of an outcome unit in an artificial neural network is conventionally a function of the input received from incoming connections (see highlighted connections in Fig. 1a), also called *net input*. The most simple version of a net input function $ne{t_{j}^{t}}$ of an outcome *j* is the sum of weights $v_{xj}^{t}$ of all cues *x* present at the current time *t* to an outcome *j* (McCulloch & Pitts, 1943; e.g., also used in Rescorla & Wagner, 1972; Rosenblatt, 1962; Widrow & Hoff, 1960):

2 $$  ne{t_{j}^{t}} = \sum\limits_{x\in cues(t)} v_{xj}^{t} $$

In neural network architectures, the net input to an outcome is then further transformed by an activation function, which can significantly influence the learning behavior of the network. At this point, error-driven learning models diverge. For example, a so-called heavy-side step function which equals zero below a specific threshold and one above this threshold can implement the assumption that outcome units can only be on or off (McCulloch & Pitts, 1943; Rosenblatt, 1962). Widrow and Hoff (1960) and Rescorla and Wagner (1972) assumed that the input of an outcome unit would not be transformed for the error calculation (or transformed with a linear identity function). Finally, modern neural networks usually assume a nonlinear activation function which allows to capture non-linear structures in the input (e.g., Rumelhart et al., 1986). With our aim of simplifying the error-driven learning model as much as possible, we however opt for the linear identity function (equal to no transformation) in line with Widrow & Hoff’s, 1960; Rescorla & Wagner’s, 1972 original model. We therefore define the activation as equal to the net input:

3 $$  ac{t_{j}^{t}} = ne{t_{j}^{t}} = {\sum}_{x\in cues(t)} v_{xj}^{t} $$

The formulation of the learning rule for the actual error-minimization process also differs slightly between different suggested error-driven learning models. The version with the least free parameters is the Widrow-Hoff rule commonly referred to as the *delta rule*. (In the following Section “Mathematically similar learning rules” we will discuss mathematically similar formulations and alternatives to this rule, especially also the differences with the Rescorla-Wagner learning rule which is mostly used in the context of current research employing two-layer error-driven learning networks.)

In a network with discrete cue and outcome units with activation boundaries between 0 and 1, the delta learning rule differentiates between three possible learning situations to calculate the weight difference ${{\varDelta }} V_{ij}^{t}$ at every time step *t*:

4 $$  {{\varDelta}} V_{ij}^{t} = \left\{\begin{array}{lll} 0   & \text{, cue \textit{i} absent} \\ \eta(1 - ac{t_{j}^{t}})   & \text{, cue \textit{i} and outcome \textit{j} present} \\ \eta(0 - ac{t_{j}^{t}}) & \text{, cue \textit{i} present but outcome \textit{j} absent} \end{array}\right. $$

In the first of the three cases, when a cue does not occur, no weights are updated. The second case describes the situation when a cue and an outcome co-occur. In this case, weights are adjusted according to the error between the maximal possible weight value 1 and the current activation of the outcome *j* modulated by a learning parameter *η* (per default, we suggest to set *η* = 0.01, as in: Baayen et al., 2016b; Hoppe, van Rij, Hendriks, & Ramscar, 2020; Ramscar et al., 2010). In the third case, the weights from the current cue to every *absent* outcome in the network are adjusted following the error between the activation of the absent outcome *j* and 0.

The main characteristic that makes the delta rule discriminative is that weights from single cues to single outcomes are always updated as part of a system of weights which influence each other (see Fig. 1a), in particular, including the weights from all currently present cues to all outcomes (present and absent). On the one hand, all present cues in a learning event influence each other’s weight adjustment in the activation term ${\sum }_{x\in cues(t)} v_{xj}^{t}$ (see Eq. 3 and Fig. 1c). Together with the fact that 1 defines a maximal weight value, which restricts the error term $1-ac{t_{j}^{t}}$ in Eq. 4, this leads to the dynamic of cues competing with each other for predicting a specific outcome, or in other words, for their share of the maximal weight value. On the other hand, weights from one cue are always updated respective to all outcomes in the network, also the outcomes that are absent in the current learning event (see Fig. 1c).

What follows from updating the whole network after every learning event is that the delta rule can *associate* and *dissociate* cues from outcomes (see Fig. 1c). Association, the process of increasing weights from cues to outcomes, is a process mainly driven by positive evidence, thus, when cues and outcomes co-occur. However, perhaps more importantly, the delta rule also allows for dissociation of cues from outcomes. First, the limit 1 on weight increase creates the need to down-regulate overshooting weights. Second and more importantly, the third case of Eq. 4 decreases weights when cues wrongly predict an absent outcome, thus, when the learner is confronted with negative evidence. As a consequence of this interplay between association and dissociation, weights will not only depend on how often a cue occurs with an outcome but also on how often it does not occur with that outcome. Learning is therefore not only depending on cue and outcome frequency but also on how predictive, or in other words, how *informative* a cue is for an outcome (Gallistel 2003, 2002, Rescorla 1988, 1968; “information” is here used as defined by Shannon 1948), meaning how much a cue can reduce the uncertainty about the outcome and so contribute to a maximal discrimination of cue structures.

In sum, when analyzing error-driven learning in such a simplified framework we can observe its discriminative nature: input is processed incrementally in order to directly learn predictive structures in the environment with the aim of fully reducing uncertainty. To simulate this process, it is sufficient to use a two-layer feed-forward network in which outcome units are activated directly by summing up incoming weights, without any further transformation. The weights are learned in an error-minimization process which incrementally updates the whole network by applying two mechanisms: first, outcome competition not only increases connections between co-occurring items or events, but more importantly also simultaneously decreases connections between non co-occurring items or events; and second, cue competition evaluates the informativity of single cues relative to all currently present cues. Together these two mechanisms ensure that at any point in time ideally only one unique outcome (or one unique set of outcomes) is expected while all others are discarded. (However, as we will illustrate in more detail in Section “?? ??”, depending on the learning problem at hand, this *ideal* goal is often not reached.) In particular, we suggest that this minimal error-driven model is not only sufficient but also most suited to study the basic mechanisms of learning because the minimized parameter space decreases the risk of confounding the underlying reasons for any observed behavior of the model.

### Mathematically similar learning rules

It is important to note that the Widrow-Hoff learning rule or delta rule presented in Eq. 4 is mathematically equivalent to linear regression (Evert & Arppe, 2015; Gluck & Bower, 1988) and very closely related to logistic regression (Evert & Arppe, 2015), which uses a logistic activation function instead of an identity activation function.

All of these accounts provide a least-squares solution (minimizing the squared differences between the target outcome activation 1 or 0 and the actual outcome activation summed over all training trials) that an incremental learner will fluctuate around or asymptote towards. In cases in which the learning trajectory is not of interest, there are several ways to directly calculate this solution or the equilibrium state (see e.g., Danks, 2003; Evert & Arppe, 2015).

The delta rule is also very closely related to the Rescorla-Wagner learning rule (Rescorla & Wagner, 1972), which comes, however, with some additional parameters. Because this rule is often referred to in the context of simple two-layer error-driven learning networks, we shall briefly discuss why the simpler delta rule should be preferred over the Rescorla-Wagner learning rule when studying basic error-driven learning. The Rescorla-Wagner learning rule makes several additional assumptions, which are implemented in additional parameters:

5 $$  {{\varDelta}} V_{ij}^{t} = \left\{\begin{array}{lll} 0   & \text{, cue \textit{i} absent} \\ \alpha_{i}\beta_{1}(\lambda-ac{t_{j}^{t}})   & \text{, cue \textit{i} and outcome \textit{j} present} \\ \alpha_{i}\beta_{2}(0 - ac{t_{j}^{t}}) & \text{, cue \textit{i} present but outcome \textit{j} absent} \end{array}\right. $$

First, in this formulation the upper target value of an outcome’s activation is not restricted, but defined by a more general parameter *λ*. As it is applied to all outcome units, *λ* acts only as a scaling parameter and can therefore be generally set to 1.

Second, in contrast to the general learning rate *η* in the delta rule, the Rescorla-Wagner rule allows for a more detailed specification of a salience parameter *α*_*i*_, which can vary by cue, and for two learning rates, one for positive evidence (case 2 of Eq. 5), *β*_1_, and one for negative evidence (case 3 of Eq. 5), *β*_2_ 3.

A general problem that these salience parameters raise is that the concept of “salience” is typically ill-defined in the literature (e.g., in linguistics: Boswijk & Coler, 2020). In particular, because salience is often conceptualized as a property of a stimulus which makes it “stand out” from its surrounding context, the whole idea of salience is conflated with discriminability. Since the point of delta-rule learning is to determine cue weights in context — which makes some cues more salient than others — from a theoretical perspective, adding a salience parameter to the delta rule appears to be at best unparsimonious and at worst circular. For example, the parameter *α*_*i*_ has been used to account for overshadowing (Rescorla & Wagner, 1972). However, in modeling blocking, which is essentially a special case of overshadowing, this effect is more parsimoniously explained by previous learning, which is reflected in the weights between cues and outcomes in the network (see Section “Cue competition”), an explanation that provides more insight about underlying processes than fitting the effect by tuning an *α*_*i*_ parameter. Similarly, the use of different *β*_1_ and *β*_2_ has been mainly motivated by the assumption that the presence of outcomes is inherently more salient than the absence of outcomes, which is why originally Rescorla and Wagner (1972) set *β*_1_ = 0.2 and *β*_2_ = 0.1. While this assumption is broadly sensible from a discriminative perspective, given that it is difficult to determine reliable cues for an outcome which occurs in many situations and that most outcomes are more frequently absent than present, there are also situations in which the absence of an outcome is more salient, for example, when it is more frequently present than absent (McKenzie & Mikkelsen, 2007). This suggests that assumptions about salience in regard to the representations employed in learning models need to be made very carefully, and that other options, such as attending more closely to the learning task and the inevitable prior experience of learners may be more preferable than simply tweaking an effect that the model itself is supposed to capture. Overall, we suggest that, on the one hand, simple error-driven learning models can be usefully employed to investigate the still elusive concept of salience by manipulating the training data or input representations. On the other hand, when the salience of elements of a representation cannot be captured in this way — perhaps because of the effects of evolution on learners’ perceptual system — it might be best to use more detailed models that explicitly attempt to capture these effects as opposed to hard coding them in a simpler more general model.

It is worth noting that, probably, many of the original modifications to the delta rule associated with the Rescorla-Wagner learning rule might be best understood in terms of the limited options available to modelers when it was first proposed. At that time, there were few alternative formal models of learning, and computational resources were extremely limited. Accordingly, modelers were forced by necessity to work only with simple learning mechanisms and very simple representations. Further, when iterations were still often calculated by hand, adding these parameters clearly simplified the computational complexity of actually running simulations. Simply assuming that one cue was more salient than another could allow a modeler to avoid the task of reconstructing the learning trajectory that actually led to that salience. By contrast from a modern perspective, the costs associated with processing more complex input representations and training regimens using the delta rule (which can exert an enormous influence on model outcomes, Bröker & Ramscar, 2020) are now relatively trivial, and a range of models with different strengths and weaknesses now exists for modeling tasks where use of the simplified delta rule may be inappropriate (this is discussed in detail further below). Thus, rather than seeking to explain learning phenomena by constantly modifying the same simple learning mechanism, when this mechanism is not suited to a task, modelers today have the option of selecting different learning algorithms and/or architectures that might be more appropriate to the phenomena in question.

In conclusion, we would suggest that these free parameters actually get in the way when it comes to using simple error-driven learning models in research. If this suggestion is followed when using the simple delta rule (Eq. 4), it further follows that all learning dynamics can be attributed to the core mechanism, which comprises the processes of association, dissociation and cue competition, and to the underlying frequency distribution of cues, outcomes, and learning events. In the following section we will now isolate the main learning dynamics that result from the basic error-driven learning mechanism. However, we need to foreshadow: the employment of the delta learning rule is not sufficient for an error-driven learning network to show the dynamics of discriminative learning. Rather, discriminative learning unfolds from an interaction between the learning rule and the structure of the input (see Section “Cue and outcome representations”).

## Learning dynamics

Having presented a simplified network architecture and learning mechanism, we next turn our attention to the details of basic error-driven learning dynamics. In the following we will lay out how they arise from the basic setup we have described in the previous section.

In order to understand the basic learning dynamics in an error-driven learning model, we need to, first of all, return to our main assumption from the previous section that error-driven learning serves to minimize uncertainty about upcoming states of the world. The prediction error is reduced maximally, when the summed weights of a set of cues approximates 1. Consequently, cues have to compete for their share of the limited outcome activation, a process called *cue competition*. However, full certainty about outcomes cannot be achieved in every situation, due to ambiguous cues or missing information. For example, when seeing a bird in a tree (without having access to binoculars), we only can use the bird’s size to classify the bird, which is often not a reliable predictor. In this case, we would be more likely to conclude that this bird was a Great Tit rather than a Coal Tit, because the first species is much more commonly seen in the Netherlands than the latter. Thus, in situations in which uncertainty cannot be fully decreased, the best alternative is to expect outcomes according to their probability under the current circumstances. In error-driven learning, the updating of weights to absent outcomes (see Eq. 4, case 3) leads to *outcome competition*, which makes sure that the most probable outcome in a situation is favored.

Importantly, situations in which only cue competition or only outcome competition play a role, are very unlikely. Usually we are confronted with situations in which these two mechanisms interact. However, to explain the two dynamics, we will first discuss cue and outcome competition separately and then investigate how they work together in error-driven learning.

Thereafter, we will discuss how the qualitative difference between cue and outcome competition gives rise to asymmetry effects and touch on the temporal dynamics in error-driven learning models.

### Cue competition

**Fig. 2:**
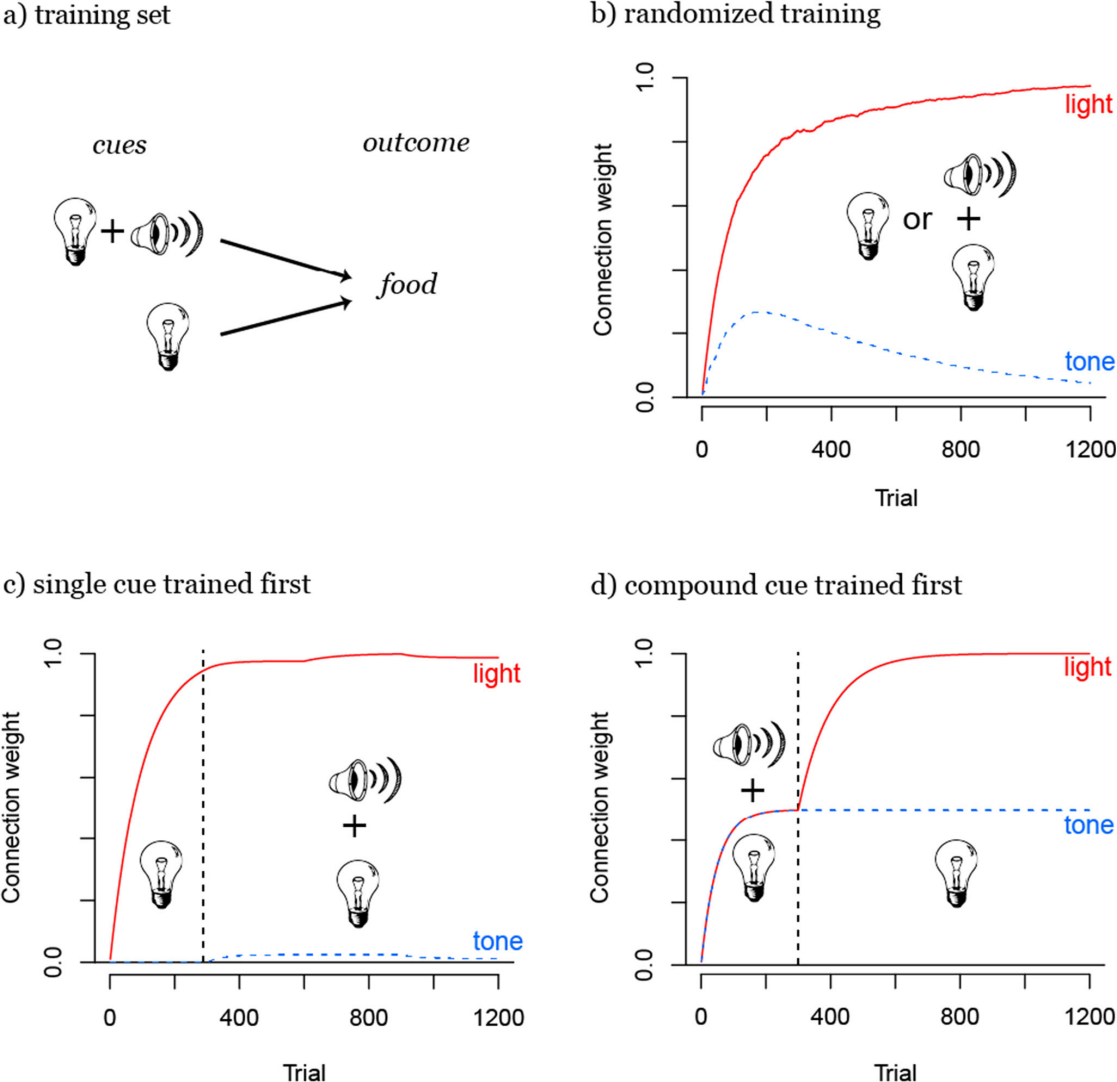
Illustration of the cues and outcome in Kamin (1969) blocking paradigm (a). During randomized training the weight from the more frequent light cue to the outcome *food* is increased until light completely predicts food by itself (b). This effect is amplified when the light is trained first by itself to predict food (c). While in b) the tone can temporarily increase its weight, it almost can’t increase its weight in c). When the compound cue consisting of light and tone is trained first (d), the weight of the tone cue stays constant (until a new training regimen, e.g. as in b) would be applied)

Cue competition occurs when multiple cues appear with the same outcome. A very simple example of cue competition is Kamin’s (1969) *blocking paradigm* in which rats were trained to expect a shock by either presenting a light or presenting a tone (“noise”) together with a light before a shock. Kamin observed that after the rats had learned to expect a shock after a light, they would not subsequently learn the predictive value of a new cue, the tone, appearing together with the light. In the following, we will use this example (replacing the shock with food, see Fig. 2a) to illustrate the dynamics of cue competition and to analyze how these dynamics change under different training regimens.

First of all, cue competition serves the function of maximizing the activation (i.e., the expectancy) of an outcome given every possible set of cues by optimizing the cue weights to this outcome. In our example, this means that the weights of the cues tone and light to the outcome food will be optimized such that both the expectancy of food given only the light and the expectancy of food given the light together with the tone will be maximal.

This optimization process entails that whenever a set of cues appears together with an outcome, the cues within the set compete for their share of the prediction of this outcome depending on how informative the single cues are about the outcome averaged across all learning events. Crucially, this competition arises from the fact that the weight update for each single cue is calculated proportionally to the activation, which is the sum of the weights of all currently present cues. Therefore, the magnitude of the weight adjustment of each single cue is affected by the weights of all other cues it co-occurs with. If one of the cues enters the competition with a high weight value, because it appears more frequently with the outcome than the other cues, the weight update will be small. As a result, the other cue(s) in the cue set will never be able to reach a similarly high weight. This happens for example in situations in which one cue appears more frequently with the outcome than the other cues, such as the light which is twice as frequent as the tone in the blocking paradigm (see Fig. 2b).

If the frequencies of the cues presented to a model are held constant, the effect of cue competition can be modulated by the temporal sequence of training trials. In the blocking paradigm this can be illustrated by observing how the training sequence influences how well the less frequent cue in the compound is learned, in our case the tone. First of all, the classic blocking effect (see Fig. 2c), where this cue is completely blocked, occurs when the light is pretrained until it fully or almost fully predicts the outcome by itself. Then, no share of the outcome activation is left for the tone in the compound with light and the tone becomes almost completely redundant. However, when training trials are completely randomized (see Fig. 2b), the tone will carry part of the outcome prediction at first and only over time, the overall more frequent light cue will fully predict the outcome by itself. Finally, when the classic blocking training sequence is reversed such that the compound cue is trained first (see Fig. 2d), the tone will, in theory, not decrease^4^ its weight when later the single light cue is trained (until light and tone would appear again as a compound cue). This illustration shows how the training sequence can influence cue competition and that it can be worthwhile to study the learning effects over time, an issue that we will come back to again later.

**Fig. 3:**
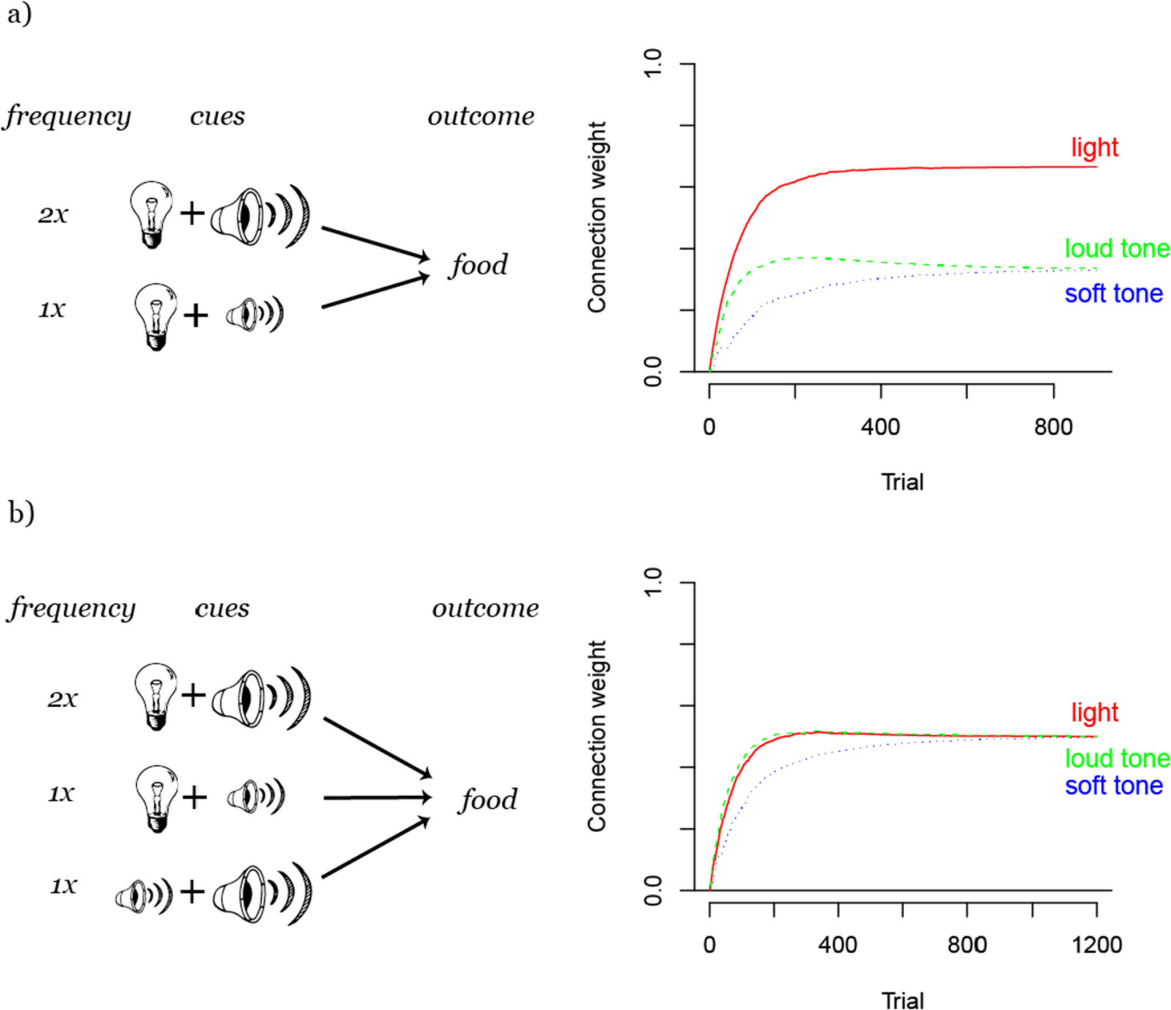
Different examples of cue competition. a) shows how frequency only determines weight differences *within* sets of cues, as the more frequent loud tone develops the same weight to the outcome *food* as less frequent soft tone. b) illustrates how frequency effects in cue competition can be canceled out by the structure of cue interactions. Here, every cue interacts with every other cue, which results in all cues having the same weight despite their different frequencies

In the previous example, frequency determined the outcome of cue competition as a function of the order in which the cues were presented. However, it is crucial to see that frequency only matters within sets of cues. To illustrate this important point, consider another example: a light cue is either paired with a loud or a soft tone preceding food, with the former set of cues being more frequent than the latter (Fig. 3a). After randomized training, the light will be the strongest and also the most frequent cue, however, both tones, the more frequent and the less frequent one, will have the same weight. In this example, not the different frequency of the two tones determines the learned weights but the identical way in which they compete with light within the set of cues.

These two examples (see Figs. 2 and 3a) serve to illustrate some basic mechanisms of cue competition: first, only cues that co-predict the same outcome as a set can compete with each other; second, within these sets, frequency of occurrence with the outcome determines which cue will develop the strongest weight; third, temporal organization of training modulates this effect, as cue competition is temporally restricted until the outcome is maximally predicted by all sets of cues. With these mechanisms cue competition can identify the most relevant cues to be able to fully predict an outcome across all possible situations (i.e., cue set - outcome occurrences).

Still, the dependencies in cue competition can quickly become very complex. Figure 3b shows what happens when not only the two different tones interact with light independently but also when they interact with each other. In that case, when all cues interact with each other, all frequency effects vanish and all cues develop the same weight to the outcome because all cues influence each other to the same extent. Hence, how cues interact with each other can take precedence over the different cue frequencies and in this way cue competition can even cancel out frequency effects.

### Outcome competition

Thus far, we have only considered situations in which there can be multiple cues but only a single outcome. Only in such cases can we observe pure cue competition (i.e., in the absence of effects resulting from the competition of outcomes). To observe the opposite case, pure outcome competition, we need to construct a situation in which there is only one cue, but multiple outcomes (note that these are highly idealized examples).

**Fig. 4:**
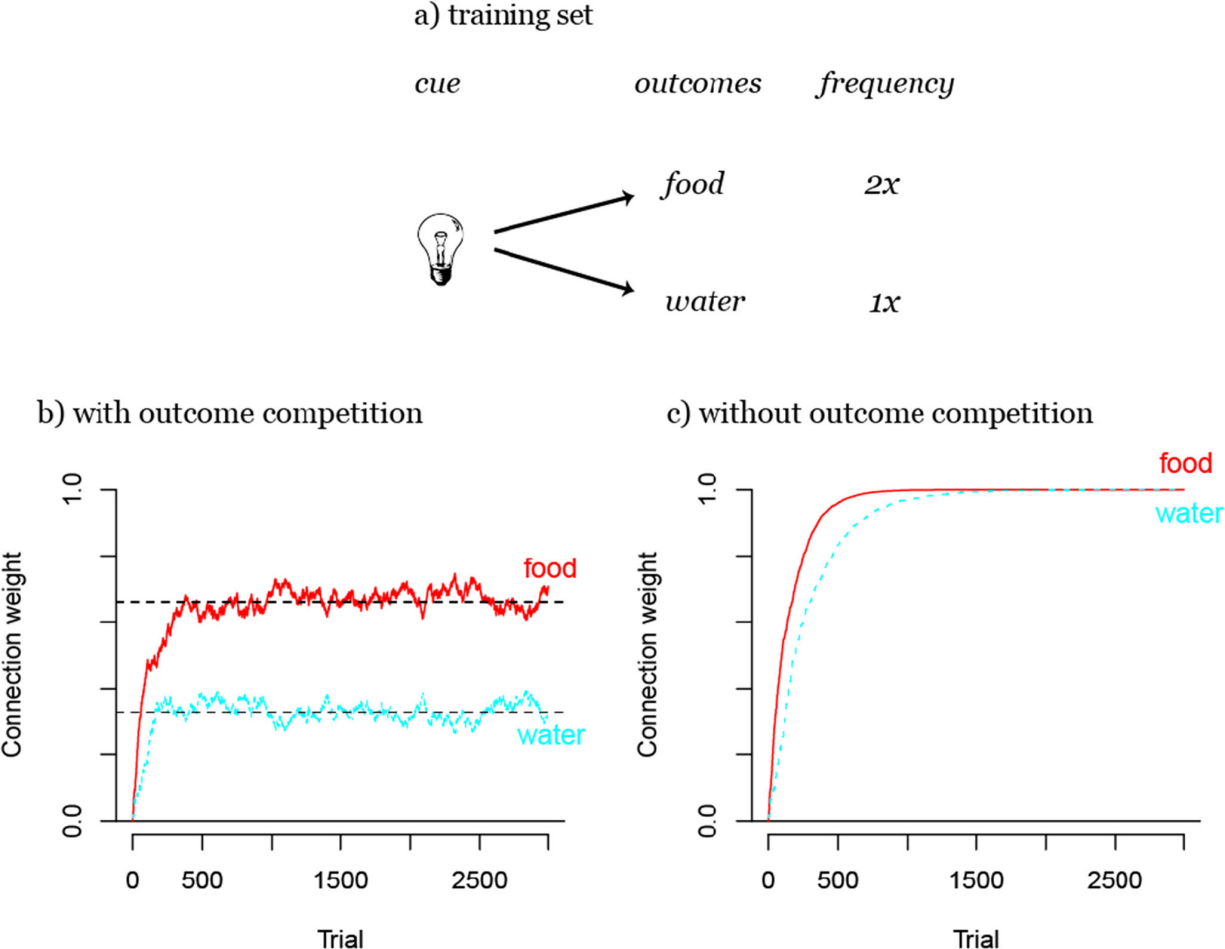
Illustration of outcome competition. In situations with less cues than outcomes (as in a), not all outcomes can be fully predicted. In that case, the updating of absent outcomes as in case 3 of Eq. 4, leads to the learning of conditional probabilities of outcome given a cue. Here, food is twice more likely to occur after the light than water (b). Without this mechanism (for illustration purposes), the single weights will both increase to the activation limit of 1 (c), a result which theoretically violates the aim of maximizing certainty of outcome predictions

When multiple outcomes are being predicted by one or only a few cues, a complete optimization of outcome activations with the aim of full uncertainty reduction will not be possible as in the previous examples. One cue cannot *fully* predict more than one outcome as would be the hypothetical aim in an example where a light cue predicts both food and water delivery (see Fig. 4a). While it seems to be intuitively possible to predict two outcomes from one cue set, this does not comply with the assumption that the aim of learning is to maximize the certainty with which an outcome can be expected. Thus, in such situations a mechanism is needed which maximizes the likelihood of choosing the correct outcome. This is exactly the objective of outcome competition: it approximates the conditional probabilities of outcomes given a cue (Ramscar, 2013; Ramscar et al., 2010).

Outcome competition in error-driven learning results from the updating of cue weights to absent outcomes (case 3 of Eq. 4). This mechanism decreases the weights from all currently present cues to all outcomes that are absent in a specific learning event. Figure 4b shows how this results in learning conditional probabilities of all outcomes given the present cue. Here, light predicts food two thirds of the time and water one third of the time. If the updating of absent outcomes is disabled, the weights to both outcomes, food and water, will rise to 1 as shown in Fig. 4c, a learning outcome which would, again, contradict the aim of maximal uncertainty reduction. Furthermore, Figure 4 shows that outcome competition reduces weights more easily than cue competition, which can reduce weights only if the maximal outcome activation is reached. The weight development with outcome competition (see Fig. 4b) appears therefore less stable than without outcome competition (which we removed from the learning algorithm for illustration purposes as described in A; see Fig. 4c). Outcome competition is thus an inherently different process than cue competition. While cue competition depends on the predictive value of cues, outcome competition depends entirely on the distribution of a set of outcomes relative to a set of cues.

### Interactions of cue and outcome competition

While it is important to note that learning should always be considered in the context of a system, we should also acknowledge that the “systems” presented as examples in the previous section, are far from ’realistic’. However, limited models like these aim to capture sub-parts of larger learning systems in order to make important local interactions in the overall learning process comprehensible. Accordingly, after having illustrated the isolated mechanisms we will now focus on how cue and outcome competition interact.

**Fig. 5:**
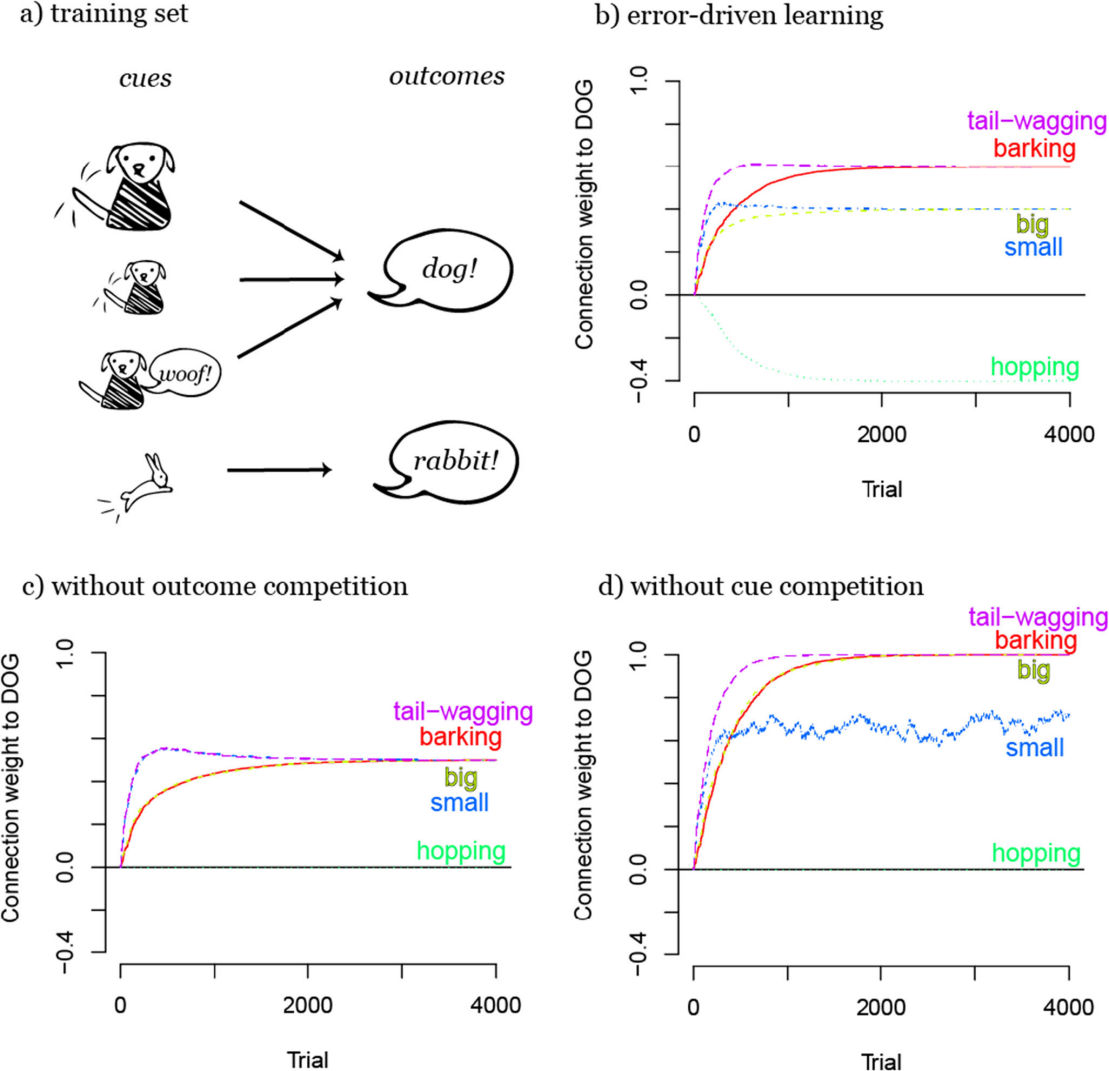
Illustration of the interaction of cue and outcome competition in dog-rabbit example 1. In this example, the weights learned with full error-driven learning (b) show that species-specific features (e.g., *tail-wagging*) are more relevant for species discrimination than shared features (i.e., size). When outcome competition is turned off during learning (c), the model does not discover that size is a feature dimension shared between the two species and cue competition leads to the same weights from all features (as in Fig. 3b). When cue competition is turned off during learning (d), weights correspond to the conditional probabilities of the label, here, “dog”, given a feature (small has a lower weight because in some cases it also precedes the label “rabbit”)

Let us consider an example which illustrates the interaction of cue and outcome competition: learning to discriminate different animals from each other. In Fig. 5, learning the difference between dogs and rabbits is simulated: in this example (*dog-rabbit example 1*), the learner encounters big and small tail-wagging dogs, small barking dogs, and small hopping rabbits. Note that this example is rather similar to the light-tone example in Fig. 3b, except that this example includes a second outcome (see Fig. 5a).

To illustrate the contributions of both cue and outcome competition in this example, Fig. 5 compares the weight development during error-driven learning as defined in Eq. 4 with weight development when either cue or outcome competition is turned off during learning (see A). After training with normal error-driven learning (see Fig. 5b), dogs are discriminated by the cues *tail-wagging* and *barking* and rabbits by *hopping*. Size (as captured by the cues *small* and *big*), however, is learned to be an overall less informative cue dimension. Remarkably, the cue *barking* is learned to be more predictive for dogs than *being small*, although small dogs are defined to be more frequent in this example (see Fig. 5a) than barking dogs. As the cue *small*, however, also appears with the outcome *rabbit*, this cue is overall less useful as compared to the cue *barking* to discriminate dogs from rabbits — which is correctly captured in the learned weights when the full error-driven learning mechanism is applied.

Figure 5c shows the weight development when outcome competition is turned off during learning by skipping the updating of absent outcomes, thus the entire third case of Eq. 4. In this case, the weight development resembles the light-tone example in Fig. 3b. This shows that without outcome competition, cue competition optimizes weights per outcome, but not across outcomes. Therefore, the simulation does not pick up on the fact that *small* is a cue which is not discriminating well between rabbits and dogs and that it should therefore have a lower weight than other discriminating cues, for example *barking*.

On the other hand, Fig. 5d shows weight development during learning without cue competition by allowing each weight to independently reach a limit of 1, as opposed to restricting the *sum* of weights of all cues currently present to 1 (as in Eq. 3). Without cue competition, outcome competition makes the weights mirror the conditional probabilities of the outcomes given the single cues. Here, the cue *big* develops an equally high weight as *tail-wagging* and *barking* as they are all predicting a dog with full certainty. The cue *small* however, predicts a dog only in two out of three cases.

Hence, as opposed to learning without outcome competition, learning with enabled outcome competition takes into account how cues appear with other outcomes in the network, such as the cue *small* appears with both outcomes, *dog* and *rabbit*. Learning without cue competition differs from learning according to the full error-driven mechanism (see Fig. 5b) in that the cue *big* will also be disregarded as a predictive cue with error-driven learning. Crucially, this happens although, evaluated by itself, it is fully predictive for the species discrimination. Yet, in contrast to assessing the predictive value of cues in isolation, the full error-driven learning mechanism discovers which cue dimensions are informative as a whole - here for example both *big* and *small* are learned to be uninformative. In particular, if cues are completely complementary, such as in Fig. 5 where every cue set contains a size cue (either *big* or *small*), they will develop the same weight and will be treated by the system as one dimension (see also Fig. 3a where the visual dimension is learned to be more reliable than the acoustic dimension). This shows once more how error-driven learning is a process which is influenced by and acts on the whole system of cues and outcomes over time and is never processing events in isolation.

On its own, cue competition can serve to compare cues within sets of cues regarding how well they can predict a specific outcome, whereas it is only together with outcome competition that it serves to maximize the outcome activation in real-world learning situations in which cue sets need to be discriminated from each other based on their occurrence with *different* outcomes. Accordingly, only the two processes working in conjunction form a *discriminative mechanism* which allows learning to find the cue dimensions which are most informative given a whole system of predictive relations.

### Asymmetry effects

The qualitative differences between cue and outcome competition predict an important characteristic of error-driven learning - it is potentially *asymmetric*, depending on the ratio of cues and outcomes (Ramscar, 2013; Ramscar et al., 2010). Intuitively, this already follows from the assumption that error-driven learning is prediction-based, as predictions are inherently asymmetric. Indeed, behavioral research has shown that changing the presentation order of cue and outcome stimuli in a task changes learning, e.g., human learning of visual categories (Ramscar et al., 2013, 2010) and various linguistic categories (e.g., Chinese tones Nixon (2020); lexical stress, Hoppe et al., 2020; number words, Ramscar, Dye, Popick, & O’Donnell-McCarthy, 2011; or noun class, Ramscar, 2013).

**Fig. 6:**
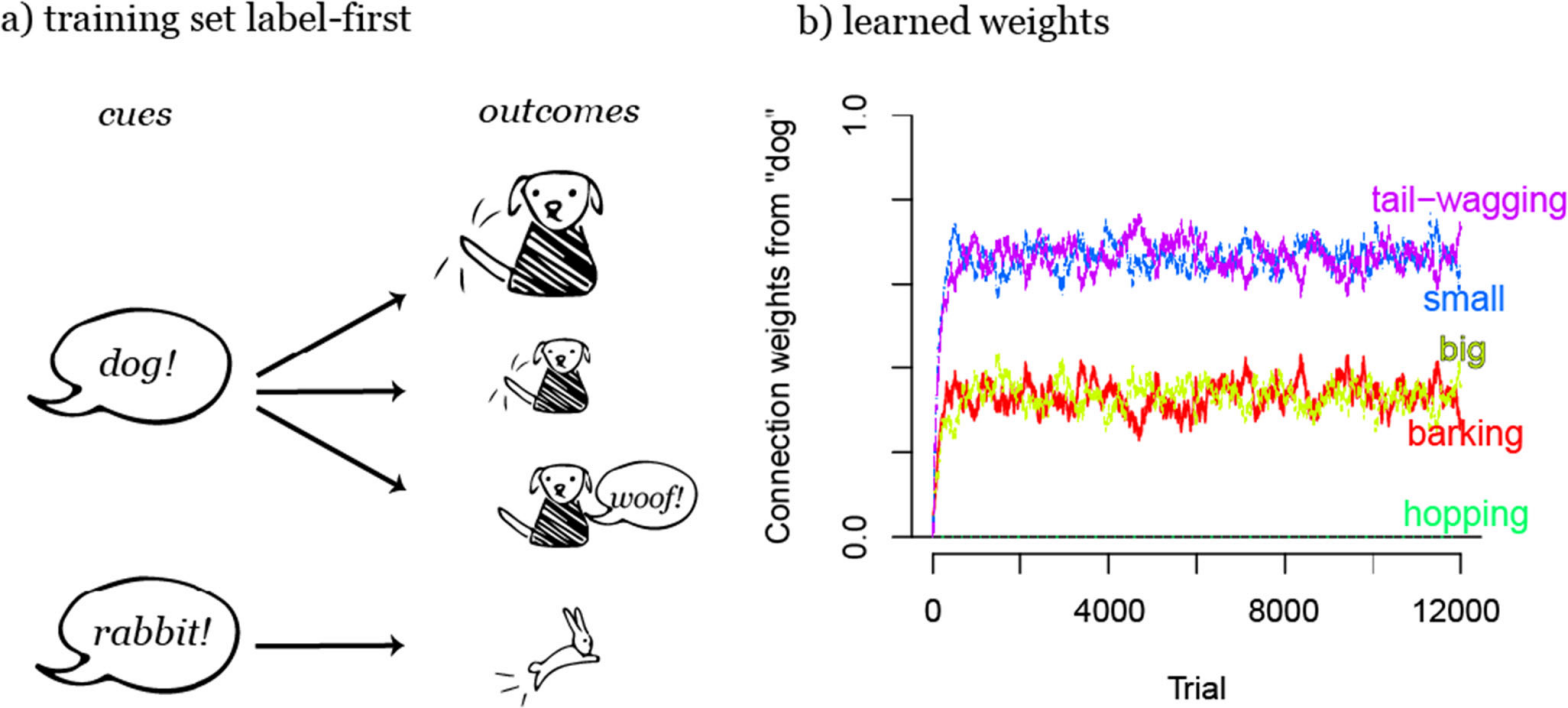
Learned weights after label-first training mirror conditional probabilities of features given a label (in this case, “dog”). Here, features that are less frequent in dogs (*barking* and *big*) receive a lower weight than features that are more frequent in dogs (*small* and *tail-wagging*). This differs from weight development in object-first training (Fig. 5), where weights correspond to the relevance of features for discrimination (in that case, size features are less relevant than the other features)

If we take a task, such as the dog-rabbit example in Fig. 5 and switch cues and outcomes, the task changes and with it the learning (compare Figs. 5b and 6b). The first cue-outcome order, when objects precede words (*object-first*, comparable to feature-label in Ramscar et al., 2010; or postmarking in Hoppe et al., 2020), simulates a learner who has to decide whether a specific animal is a dog or a rabbit (Fig. 5a). The second cue-outcome order, when labels precede objects (*label-first*, comparable to label-feature in Ramscar et al., 2010; or premarking in Hoppe et al., 2020), simulates a learner who has to decide which animal a speaker refers to when saying either “dog” or “rabbit” (Fig. 6a). The difference in weight development between these two learning situations illustrates again the difference between cue and outcome competition: the learned weights clearly differ when animal features compete for labels as cues or as outcomes. When the features compete as cues for the labels, weights correspond to how relevant a feature dimension is for the categorization (Fig. 5b); when the features compete as outcomes for the labels, weights correspond to the conditional probabilities of the features given a category (Fig. 6b).

**Fig. 7:**
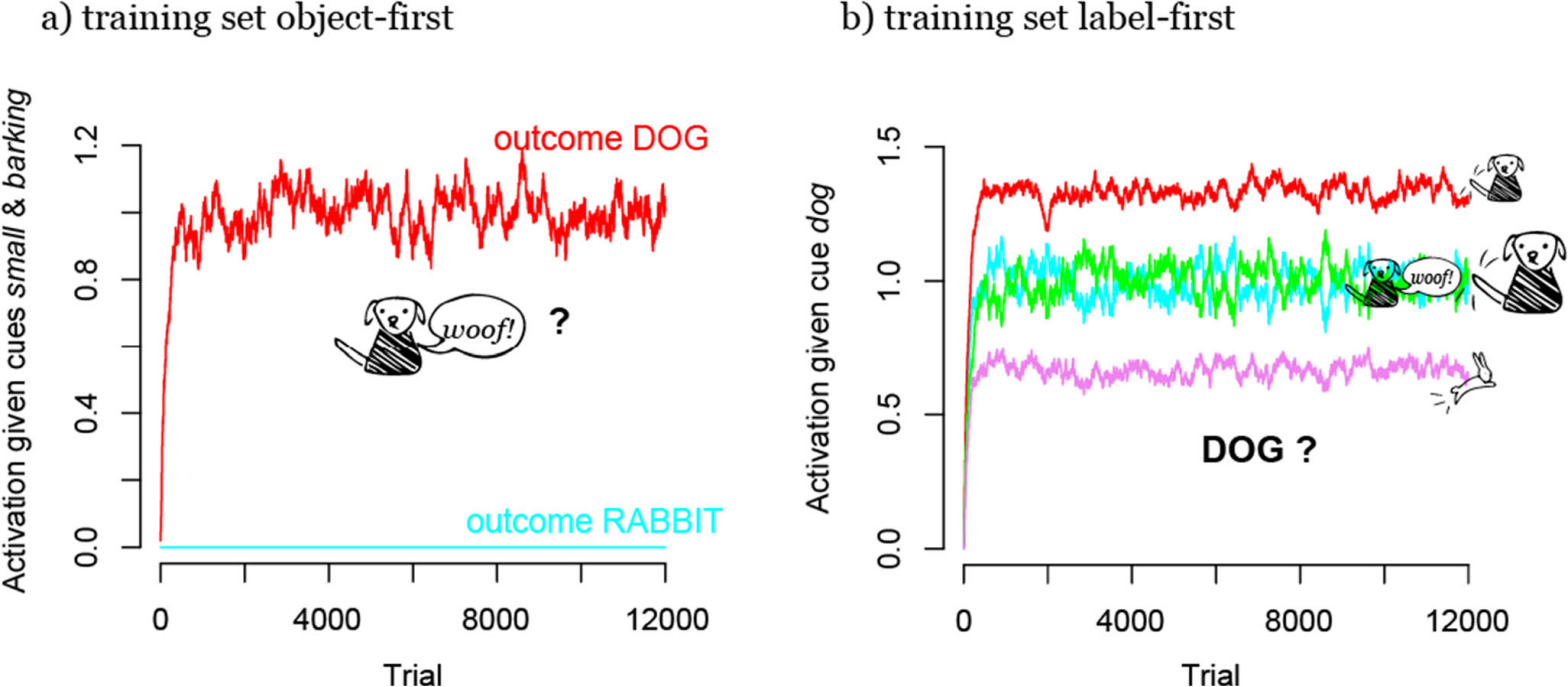
Outcome activations after a) object-first and b) label-first training on dog-rabbit example 1 (see Section “?? ??”). When objects precede labels in training (a), dogs (here shown: small, barking dogs), can be discriminated optimally: the activation of the label “dog” given a dog exemplar approaches 1 and the activation of the label “rabbit” approaches 0. However, when labels precede objects (b), optimally discriminative activations cannot be reached: given the label “dog”, dogs with most frequent features (*small* and *tail-wagging*) are expected more than dogs with less frequent features (*barking* and *big*); crucially, also rabbits are expected to a certain extent after hearing the label “dog”

Also the resulting choice behavior of a learner in this example is affected by the different cue-outcome order in the object-first and label-first situation. In both cases, a maximally discriminating learner would be completely certain about his choice. In Fig. 7a we can see that this is the case after object-first training, where the activations show that the learner expects a small barking animal always to be dog but never a rabbit (*choice probabilities* averaged over 100 simulations: *p*_*c*_(*dog*|{*small*,*barking*}) = 1,*p*_*c*_(*rabbit*|{*small*,*barking*}) = 0). After label-first training, however, predictions are not that clear (see Fig. 7b): while a small hopping animal is least predicted after the label “dog” is heard, it is still predicted in some cases (*p*_*c*_({*small*,*barking*}|*dog*) = 0.25,*p*_*c*_({*small*,*hopping*}|*dog*) = 0.17).

**Fig. 8:**
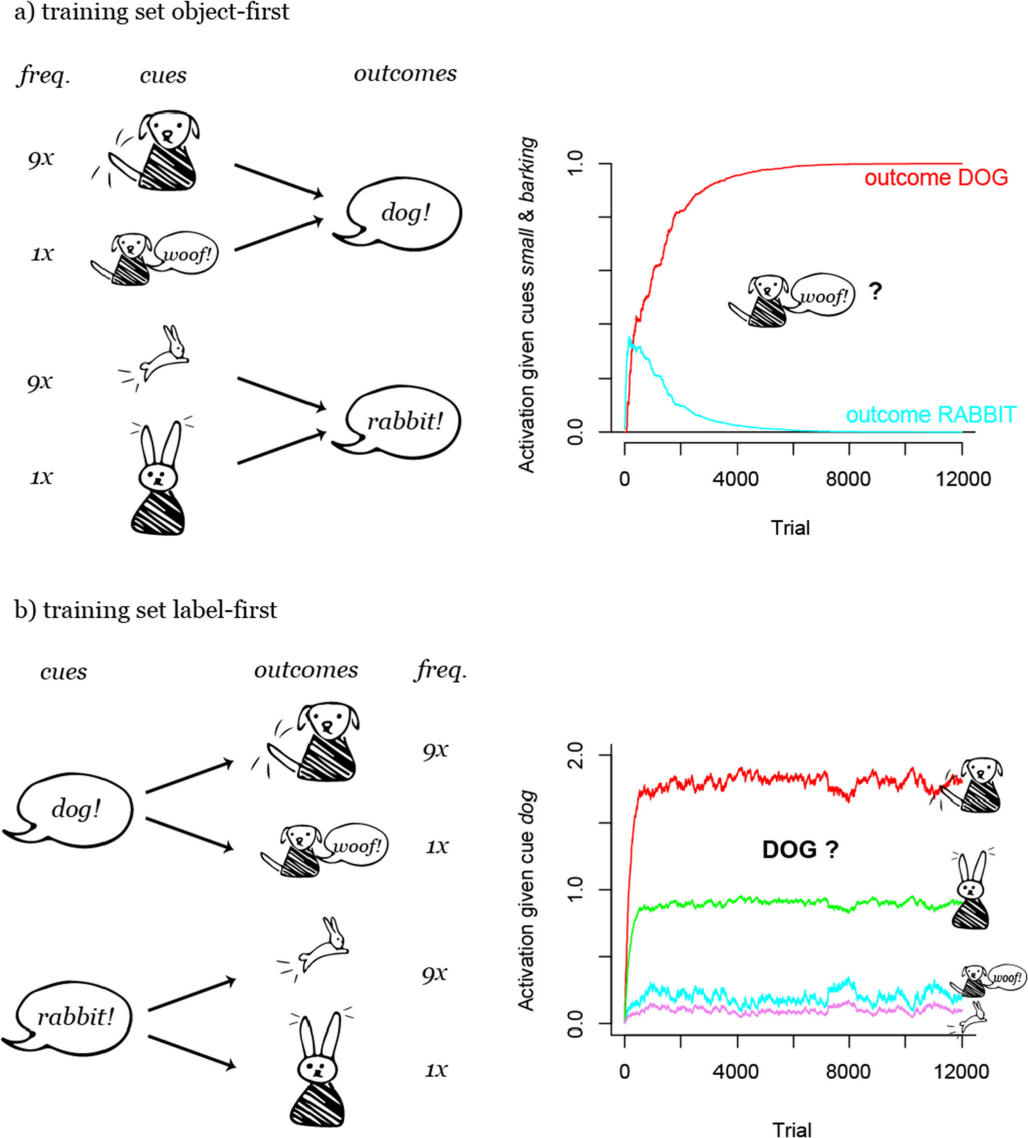
Outcome activations after a) object-first and b) label-first training on dog-rabbit example 2 (see Section “Asymmetry effects”). As opposed to example 1 (Fig. 7), misclassifications occur here after label-first training (b): after hearing a label, e.g. “dog”, low frequency exemplars of the wrong species, here, big rabbits, are expected more than low frequency exemplars of the correct species, here, small dogs. This is due to the particular kind of feature structure, in which one feature of low frequency exemplars in one species (i.e., here *big* in the species of bunnies) also occurs in high frequency exemplars of the other species (i.e., dogs)

However, the difference in learning between the two different orders of labels and objects in this example is not very large, as cue competition does not have such a strong advantage over outcome competition here. A slightly adjusted situation which is illustrated in Fig. 8 shows a more dramatic difference. In this second example (*dog-rabbit example 2*), low frequency items of one category share a feature with high frequency items of the other category (as in Ramscar et al., 2010): while dogs are mostly large animals, only a few large rabbits exist. When animal features compete as outcomes for the species labels, this leads to misclassification of low frequency items, such as big rabbits and small dogs (Fig. 8b, *p*_*c*_({*small*,*barking*}|*dog*) = 0.06,*p*_*c*_({*small*,*barking*}|*rabbit*) = 0.29). However, when animal features compete as cues, low and high frequency exemplars can be classified correctly (Fig. 8a, *p*_*c*_(*dog*|{*small*,*barking*}) = 1,*p*_*c*_(*dog*|{*big*,*tail* − *wagging*}) = 1)).

These two examples illustrate how asymmetry can affect some learning situations more than others (see experimental evidence in Hoppe et al., 2020). What both examples have in common is the asymmetric network structure which is necessary to observe an asymmetry effect in learning. In general, we can observe that when the cue layer has more features than the outcome layer, thus when the network is *convergent*, learning can be maximally discriminative because features can compete as cues for outcomes (as in Figs. 2, 3, 5). When the cue layer has fewer features than the outcome layer, thus when the network is *divergent* (Osgood, 1949; Greenberg, 1957), learning cannot be maximally discriminative and approaches conditional probabilities instead, because features compete as outcomes for cues (as in Figs. 4, 6).

This discussion of asymmetry effects in learning serves to highlight two points that are important to consider when evaluating an error-driven learning model: first, it is important to pay attention to subtle differences in temporal order when determining which entities are coded as cues and which as outcomes; second, it is important to consider the resulting network structure which can determine whether cue or outcome competition will govern the learning process.

### Temporal dynamics

In the previous section we discussed how the temporal characteristics of predictions, which are at the base of error-driven learning, transfer to the learning process. We have seen that time matters for the relation between cues and outcomes, as learning can be temporally asymmetrical. Time also needs to be considered for other parts of an error-driven learning simulation. Network weights are usually updated incrementally, which makes it possible to observe learning over time. This at the same time creates the need to consider the order of training trials, as it can have a significant effect on the learning outcome (see, e.g., the blocking effect, Arnon & Ramscar, 2012; Kamin, 1969). Ultimately, in building models, one has to decide how long a network is trained and whether the whole time course or only the point of convergence of the network is of interest in a simulation. Furthermore, time becomes a defining factor when modeling sequence learning, where detailed temporal relations can be considered during the learning process or else later during model evaluation.

#### Time course of learning and convergence

The blocking example illustrates how trial order, either blocked (Fig. 2c) or randomized (Fig. 2b), does not change the convergence point of the network but does change the time course of learning. Importantly, in a constantly changing environment, the point of convergence is just an abstract construct, which is probably never reached. Usually a learner is never a blank slate and new information will interfere with already learned information, as for example in language attrition, when a speaker switches from one dominant language to another. On the one hand, the kind of forgetting that interference in an error-driven learning network over time can produce has been framed as a weakness of the learning algorithm, the sequential learning problem (McCloskey & Cohen, 1989; Ratcliff, 1990). On the other hand, interference offers an explanation of forgetting, which we need to account for when modeling human or animal learning, without assuming decay of weights over time (e.g., McLaren & Mackintosh^2000^).

Furthermore, a closer look at some of our previous examples can reveal how learning dynamics change over time. In Figs. 3 and 5, we can observe how frequency can have an influence on learning early in training, such that more frequent cues are associated or dissociated faster from outcomes, while later in training, frequency effects are canceled out by cue and outcome competition. Considering the amount of training of the model is therefore always an important step when building or evaluating an error-driven learning simulation.

To be clear, the learning procedure according to Eq. 4 is always iterative, in line with the aim of simulating online human or animal learning. Thus, although it would be theoretically possible, weight updates are not performed in batches as it is often done in machine learning procedures to minimize noise in the learning trajectory and maximize computational efficiency. However, in cases in which the time course of learning is not of interest for a simulation, the point of convergence or equilibrium of an error-driven network, which equals the least-squares solution of the input matrix (Evert & Arppe, 2015), can be directly calculated with, e.g., Danks, (2003) equilibrium equations (implemented in Arppe et al., 2018).

#### Sequence learning

While in simulations of clearly delimited tasks (for example, animal classification) time is an additional modeling parameter which needs to be considered, it is the most crucial factor when modeling sequence learning. In our previous examples the definition of cues and outcomes was mainly dependent on two aspects: first, the task that determines the relevant outcomes; second, temporal ordering, which is crucial in defining cues and outcomes, as predictions can only be based on earlier occurring elements. The latter is also crucial for sequence learning where the task is not focused on predicting a single correct outcome or outcome set, such as, when making a medical diagnosis or choosing a word to communicate a specific meaning, but on the temporal sequence itself, such as, when mounting a bicycle or singing a melody.

One way of modeling sequence learning with a simple two-layer learning network has been suggested by Gureckis and Love (2010) who implemented an associative chain in which each element predicts the following element as an outcome, which then turns into a cue for the next following element. In its simplest form such an associative chain model has no memory, however to add short-term memory to the model, Gureckis and Love (2010) added a shift register which can add a specified number of preceding elements as cues to the current outcome.

This simple two-layer associative chain model with short-term memory turned out to be a good predictor of human performance in two sequence learning tasks (Gureckis & Love, 2010). Like human participants, the model could solve a sequential problem with low statistical complexity (sequences consisting of a concatenation of random samples of a sequence of integers, here 0 to 3, e.g., [0-1-2-3]-[2-1-0-3]-[1-3-2-0]), but both human participants and the model struggled with learning sequences with higher-order statistical dependencies (where every third element of the sequence is an XOR evaluation of the preceding two elements, e.g., [0-0-0]-[1-1-0]-[1-0-1]).

Crucially, Gureckis and Love (2010) suggest that the limited capacity of such simple models in learning complex sequences might depict human learning more realistically compared to more complex models, such as recurrent networks. Historically, the problems that associative chain models show in learning sequences with higher-order statistical dependencies have been a source of critique (e.g., Lashley, 1951) and led to a concentration on more powerful models with hidden layers and recurrence which are able to transform input representations. However, in comparing their simple associative chain model with a recurrent network (Gureckis & Love, 2010) conclude that these higher-level representations constructed by hidden layers of recurrent networks are not always used by humans: while a simple recurrent network with a hidden layer was indeed able to learn the sequence that the associative chain model could not learn, also the human participants did not pick up on the pattern. Moreover, the recurrent network was much slower in learning the sequence with low statistical complexity, in which the higher-level transformations of the network seemed to be a hindrance to solving the task. Thus, surprisingly, the simpler two-layer associative chain model clearly outperformed the more complex recurrent network in predicting human performance in the sequence learning tasks.

Gureckis and Love (2010) approach shows how two-layer error-driven learning networks can be used to model sequence learning. An interesting question that arises from the comparison of two-layer and multi-layer sequence models relates to higher-order representations. Under which circumstances should the events encountered in a learning situation be represented as undiscriminated sequential chunks, as opposed to sequences of elemental items? This is a point we will discuss in detail in Section “?? ??”.

#### Sequential processing

Another interesting example of using two-layer networks to model the learning of sequential processes is presented by Baayen et al., (2016b) who show how a network trained non-sequentially, can nevertheless still offer insight into the processes that give rise to the understanding of continuous speech.

The aim of Baayen et al.’s (2016b) work was to show how a network that does not implement word segmentation can understand continuously presented speech. Understanding speech was here simplified to activating the correct sequence of word forms when encountering a specific sequence of triphones. In order to not train the model on segmentation, the authors trained it non-sequentially creating a learning event for every sentence, in which all triphones of the sentence were given as cues and the sentences’ word forms as outcomes to the model. In the following step, they evaluated how the model would process a continuous stream of speech split up into triphones. Sequential processing, in this case, was simulated by moving a fixed-size window over the input stream of triphones, similar to the learning and evaluation procedure of Gureckis and Love (2010). In the end, although the network was trained without sequential information, it could segment the speech stream and give rise to behavior usually thought to occur at later stages of processing (after a segmentation stage), such as long-distance dependency processing.

These two examples (Baayen et al., 2016b; Gureckis & Love, 2010) show how simple error-driven learning models can simulate sequential learning and processing. For modeling these phenomena, the temporal order of events is obviously a crucial factor, however, since all learning happens in time, the incremental updating mechanism of error-driven learning can also illuminate the temporal dynamics of learning situations that may not at first glance seem either sequential or time-related. The issue of interference (see Section “?? ??”), for example, serves to highlight the fact that because learning is a process in which cue and outcome competition interact over time, temporal dynamics are a factor in almost all real world learning situations.

## Relating model outcomes to behavior

To better understand the underlying learning dynamics in a given learning situation, it is possible to directly analyze the output of an error-driven learning simulation, which is usually the weight matrix between cue and outcome layer after completed training, or a list of weight matrices for every time step. However, in order to test whether a simulation is capturing a given phenomenon, the model output has to be related to behavioral data.

The first step in determining how a given model will respond (activate an outcome) to a given input (in the form of a set of cues), is to determine the strength of support for possible outcomes, which is defined as the outcome activation (Eq. 3). This makes it possible to get the model’s response to a realistic input, thus a set of cues and not only isolated single cues, which can then be compared to the response of a human or animal learner. Calculated over all possible sets of cues and outcomes, the resulting *activation matrix* can then be used to predict specific response data, such as for example, accuracy or reaction times.

To simulate accuracy, a choice rule needs to be applied to the outcome activation vector, to derive the probabilities of outcomes being chosen as a model response to an input. As outcome activations are ordinal data, potential transformations of outcome activations to response probabilities should be functions that preserve order (Church & Kirkpatrick, 2000; Rescorla & Wagner, 1972). Transformations that have been used are, amongst others, a step function with a specific threshold (e.g., Church & Kirkpatrick, 2000), a logistic function (e.g., Gluck & Bower, 1988; McClelland et al., 1986), or the Luce choice rule (Luce, 1959; e.g., used by Gureckis & Love, 2010; Hoppe et al., 2020; Ramscar et al., 2010). Two important points to consider when calculating choice probabilities from activations are, first, that outcome activations can be negative, which needs to be corrected for to apply for example the Luce choice function (e.g., by setting negative activations to zero as in Hoppe et al., 2020, and Ramscar et al., 2010) and, second, to be aware of the choice baseline, which could differ from the baseline in the empirical data set (and which is dependent on the number of outcomes or possible choice alternatives).

To simulate empirical reaction time to an outcome for smaller data sets, the (negative) activation of that outcome given the present cues can be used directly. For larger data sets, reaction times can better be approximated by a log transformation of inverse activations (*log*(1/*activation*)) to remove skew from the data (Baayen et al., 2011). Note, however that this does not automatically extend to other types of continuous response data. Recent work by Lentz et al., (2021), for example, suggests that error-driven learning activations have to be handled differently to predict EEG data than to predict reaction time data. Hence, overall, the simulation of various kinds of behavioral responses based on error-driven learning activations is still very much work in progress.

The output of an error-driven learning model, that is, the learned weight matrix and the outcome activation matrix, has furthermore been used to derive more abstract measures than the direct simulation of behavioral responses (as, e.g., employed in Baayen, Milin, & Ramscar, 2016a; Hendrix, 2015; Milin et al., 2017). However, it needs to be stressed that the trade-off between trying to model a problem as precisely as possible and trying to derive an explicit contribution to theory (Bonini’s paradox) can also be extended to the analysis of a model’s output. While more abstract measures can be used to derive alternative predictors for behavioral data, it is also more difficult to clearly interpret them.

## Cue and outcome representations

The error-driven learning mechanism formulated in Eq. 4 is a mechanism which discriminates cues from each other by associating and dissociating them from outcomes, leading to the learning of positive, neutral or negative expectations. However, while error-driven learning, in theory, is a discriminative process, the full mechanism can only arise in a model operating on suitable cue and outcome representations which actually define a discrimination problem (Hoppe et al., 2020; Ramscar, 2013; Ramscar et al., 2011; Ramscar et al., 2010).

First and foremost we need to be explicit about the theoretical role that we assume representations to fulfill in error-driven learning models: that they are supposed to capture accessible information in the environment that can potentially be used to reliably predict upcoming items or events. This means we do not assume representations to actually mirror concrete neurobiological states of the brain, especially given the fact that “detailed, empirically grounded theories of how the brain encodes complex inputs are rare in the literature” (Bröker & Ramscar, 2020, p.1).

Given this basic assumption about the theoretical role of representations, we next need to distinguish between two different ways of conceptualizing representations on varying levels of granularity. Historically, the error-driven learning model by Rescorla and Wagner (1972) was used to observe how weights between cues and outcomes develop depending on how these cues appear with each other in compounds. For this reason only cue representations on one (not clearly defined but suggested low) level of granularity — so-called *elemental* cue representations — were given as input to the model. These elements were assumed to compositionally combine to represent stimuli combining elemental properties (i.e., the combination of stimuli A and B would be represented as {*A*,*B*}). Subsequent work, reacting to limitations of this purely elemental (or compositional) approach, allowed also novel representations for cue combinations (i.e., the combination of stimuli A and B would be represented as {*AB*}) — also referred to as *configural* cue representations (see, e.g., Pearce, 1987). Importantly, the configural conceptualization of representing cue combinations is in line with the idea of learning being a process of abstraction (Ramscar et al., 2010; Rosch, Mervis, Gray, Johnson, & Boyes-Braem, 1976): in this process, unpredictive input representations are unlearned, potentially (but not necessarily) leading to more abstract representations instead of detailed compositional representations. In the following, we therefore refer to configural representations as representations on *higher levels of abstraction* than combinations of elemental representations.

Whether (and sometimes when) associations are learned between more abstract configural representations or combinations of elemental representations, has been widely discussed in the literature (Harris, 2006; Pearce, 1987, 2002; Wagner & Brandon 2001). Importantly, from a discriminative perspective, focus is put less on *whether* but on *when* representations on different levels of abstraction are used for discrimination, as we will argue in this section. First of all, discrimination is assumed to be a process of uncertainty reduction which, beginning on high levels of abstraction that allow only for coarse discrimination leads to lower levels of abstraction that allow for more detailed discrimination (Ramscar et al.,, 2013, 2010). This idea runs counter to the elemental view which assumes that predictive structure between items or events in the environment can be uncovered starting from low levels of granularity. In addition, the aim of discriminative models is to learn the predictive structure between items or events in an environment. We will argue in this section that only a model presented with a sufficiently vast set of cues and outcome representations on different levels of abstraction can discover the relevance of specific cue representations to optimally predict the given outcomes. Hence, in this section we will first focus on the limitations connected to the definition and historic applications of the Rescorla-Wagner model based on an elemental view of the formation of associations in an environment. Then, we will sketch out how cue and outcome representations should be defined from a discriminative point of view.

Given the asymmetry in error-driven learning networks, we split this discussion up into separate considerations about cue representations and outcome representations. We will first focus on cue representations, where the main task of a discriminative model is to discover the optimal discriminative structure given a set of outcome representations. Note that the discussion of cue representations is especially important for simple two-layer networks, which cannot transform the predefined input representations, such as in networks with hidden layers or recurrence. Later, we will then focus on outcome representations, which can be seen as dictating the task and therewith the discrimination of cue representations.

### Elemental and configural cue representations

We begin with a review of the historical approach to representation in learning models which originates in the associative/compositional tradition of learning in psychology and assumes that association is a purely elemental process in which the relevant granularity levels of stimulus representation can be built up gradually starting from low levels of abstraction by combining some kinds of elemental stimuli.

Accordingly, when Rescorla and Wagner (1972) first presented their error-driven learning model it was applied to so-called elemental cue representations. The question, which levels of percepts can be classified as sufficiently “low-level” or “elemental” is never really discussed, so that we would suggest to interpret this term in a relative sense contrasting representations on higher levels of granularity. In fact, to explain learning phenomena observed in simple animal studies such as the blocking effect (Kamin, 1969) no further considerations of stimulus representation is needed. For example in simulating blocking, the combination of the blocking and the blocked stimulus coded compositionally as two separate cues successfully explains the learning dynamics (see also Section “Cue competition”). However, this kind of coding of cue configurations soon reached a limit in explaining effects with more complex, conditional relations between stimuli such as negative patterning (A → reinforcement; B → reinforcement; AB → no reinforcement, also written as A+; B+; AB- in the context of classical conditioning) or biconditional conditioning (AB+; CD+; AC-; BD-; Harris, 2006; Melchers, Shanks, & Lachnit, 2008; Miller et al., 1995).

The crucial assumption that was necessary to explain some of these basic learning phenomena, such as blocking, but that posed problems for others was that the weights of different cues are independent from each other and thus can be simply combined by summation. On the one hand, summation is a crucial concept which enables competition between cues as seen in Eq. 4, in which weights are summed up in the activation term. Empirically, findings of summation effects, for example, that the compound of two stimuli evokes a stronger response than the stimuli in isolation, are mixed (Melchers et al., 2008). While summation is mostly observed for stimuli that are clearly separable (Lachnit, 1988) or from different modalities (Aydin & Pearce, 1997; Kehoe, Horne, Horne, & Macrae, 1994), it is less observed for integral (compositely perceived) and same-modality stimuli. (We need to note here that these concepts that have been put forward to explain the presence or absence of summation effects might be equally difficult to define as the concept of elemental percepts. From a discriminative perspective, these differences between cue representations might also be connected to how relevant single representations are regarding discrimination in a current or previously experienced task.)

**Fig. 9:**
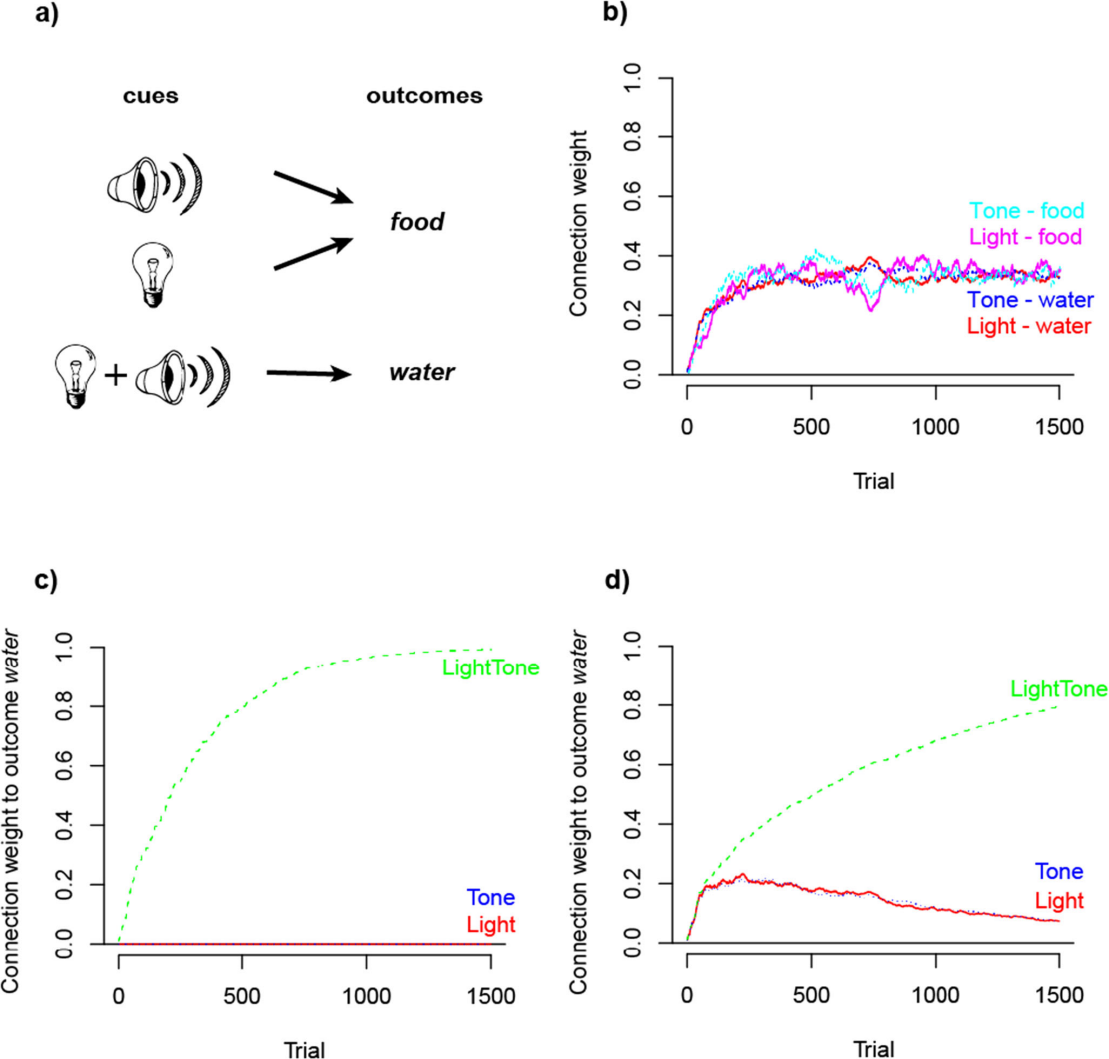
Different cue structures to model negative patterning, in which single stimuli predict a different outcome than their combination (a). When the stimulus compound is coded compositionally as a combination of its elements ({*Tone*, *Light*}), the two outcomes cannot be discriminated from each other (b). When the stimulus compound is coded by a single configural cue ({*LightTone*}), discrimination is optimal but not realistic (c). The combination of a configural cue and its elements ({*Tone*, *Light*, *LightTone*}) captures discrimination and generalization (d). See also the interactive interface in the tutorial

On the other hand, the summation principle alone makes it difficult to explain how the weight or activation given a configuration of cues could be different from the sum of its parts. Yet, for instance, this is a necessary prerequisite to explain negative patterning. Figure 9 shows an example of negative patterning, where two cues, a tone and a light, presented by themselves predict one outcome, *food*, but in combination predict another outcome, *water*. When the combination is coded elementally/compositionally (i.e., with the cue representations *Light* and *Tone*; Fig. 9b), the distinction between these two outcomes cannot be learned because the elemental cues become associated to a similar extent to both outcomes.

In reaction to the limitations of models with purely elemental cue representations, different extensions were suggested (for comprehensive reviews refer to Harris, 2006; or Melchers et al., 2008). Most importantly, many extensions include the addition of cue representations on higher levels of abstraction, for example non-compositional representations for combinations of lower-level cue representations (e.g., a cue *LightTone* that combines the cues *Light* and *Tone*). One suggestion contrasting a purely elemental approach was put forward by Pearce (1987), who suggests a purely configural approach in which cues that appear together are only represented by one configural cue omitting cue representations on lower levels of abstraction. Figure 9c shows how this can lead to very good (too good according to Harris, 2006) learning in the negative patterning problem. However, to be able to account for generalizations between cue configurations sharing lower-level elements, additional mechanisms were needed, such as a similarity measure between cue configurations. A simpler approach is to allow for both elemental and configural cue representations (as suggested by Rescorla, 1973), or in other words, cue representations on multiple levels of abstraction (e.g., used by Ramscar & Dye, 2009; Ramscar et al., 2013; Ramscar et al., 2011). Figure 9d illustrates how this approach also leads to successful learning of negative patterning, as the model learns which cue representations on which level of abstraction are informative for which outcomes, while still accounting for interference between cue combinations. One major problem with the latter approach is, however, that aiming to include all possible abstract configural cue representations in a model’s input representations quickly leads to a combinatorial explosion (Gluck and Myers, 2001).

Besides these extensions, more complex elemental models based on Rescorla and Wagner (1972) and Widrow and Hoff (1960) have been proposed, in which parsimony is traded for more explanatory power. McLaren and Mackintosh (2000), for example, refined the elemental cue representation approach by assuming continuous probabilistic sampling of “microfeatures”, which was also able to explain conditional discrimination, such as negative patterning. However, together with an added weight decay and stimulus salience mechanism, this model outsourced dynamics that could in theory be explained by simple error-driven learning. On the one hand, to avoid confounding, we suggest to always carefully weigh the advantages of such extensions that risk to come with a loss of understanding of the underlying mechanisms. On the other hand, we do not want to discourage exploring low-level cue representations (see also Ghirlanda, 2005), as depending on the problem at hand, we certainly need representations on all levels of abstraction.

A final way to include cue representations on multiple levels of abstraction into an error-driven learning model is to change the network architecture and add hidden layers to the network (e.g., Delamater, 2012; McLaren, 1993; Schmajuk, Lamoureux, & Holland, 1998). These models directly address the question of how higher-level, “configural” representations arise in a model, for example, a node in a hidden layer can represent the combination of two cues in the input layer. One advantage of this is that in this way they solve the combinatorial explosion problem, with which two-layer models are confronted. However, the disadvantage of this approach is that it is not directly observable what kind of representations are learned in a hidden layer. Therefore such models make it difficult to understand what kind of representations, i.e., on what level of abstraction, are relevant for a specific learning situation.

At this point, we need to elaborate on a principled problem with the associative approach to cue representations: its theoretical commitment to specifically defined input representations. As by definition, elemental representations on all levels of granularity can be traced back to a set of elemental representations, associative learning theories need to provide a theoretical motivation for specifying these elements. This is problematic given that the approach of trying to concretely and compositionally define input representations is inherently regressive (Ramscar et al., 2010), especially given that the goal of the endeavor of identifying neurobiologically plausible elemental input representations for such simple models is questionable considering the complex and still ill-understood myriads of networks underlying higher-level processing in the brain (Bröker & Ramscar, 2020). In contrast, within a discriminative theory the theoretical commitment to specific representations is neither necessary nor wanted, as learning models are explicitly conceptualized as tools to investigate what kind of representations are theoretically needed to predict a given set of outcomes (these are often referred to as distributed representations in the connectionist literature as, e.g., in Rumelhart, Hinton, & McClelland, 1986). Importantly, the set of hypothetically possible representations is not restricted to a particular form or level of abstraction. They are only required to conform to discriminative principles, which means that they should capture contrastive patterns in the environment, which can be of any form and on any level of abstraction.

In summary then, this discussion about elemental and configural representations in fact raises questions about the kind of levels of abstraction in cue representations that are best employed in two-layer error-driven learning models. The fact that two-layer models cannot construct higher-level representations during learning permits only one conclusion, namely that such representations have to be explicitly given to a model in order for it to veridically simulate problems, such as negative patterning. Thus in the next section we will argue that, among other factors, only if a model is confronted with cue representations on different levels of abstractions, it can make a realistic estimation about which cues on what levels of abstraction are informative for solving a given task, which should exactly be the objective of learning from a discriminative perspective. This idea is furthermore supported by previous evidence and models which suggest that cue representations on different levels of abstraction are learned to be relevant based on task demands (e.g., Dye & Ramscar, 2009; Hoppe et al., 2020; Melchers et al., 2008; Ramscar, 2013) and depending on learning experience (e.g., Arnon & Ramscar, 2012; Ramscar et al., 2013).

### Defining discrimination-friendly cue representations

All representations are wrong but some are useful. -adapted from George E. P. Box

As the foregoing discussion hopefully makes clear, there is no one right way to represent the cues involved in a learning situation, just as there is no right way to formulate a model (Box, 1976). The usefulness of specific representations strongly depends on the problem at hand and factors such as task demands, prior experience, experimental instructions and stimulus properties (Melchers et al., 2008). In fact, this is exactly the task of learning from a discriminative perspective: *learning* to represent the environment in a way that suffices for a given task (translated into outcome representations), rather than assuming a fixed set of low-level representations that compositionally combine, as it is usually conceptualized from an elemental/associative point of view (see, e.g., Harris, 2006; Smolensky, 1988). Accordingly, this makes it even more important to thoroughly consider what kind of cue structure is suited to model a given problems.

As we have noted above, although historically, error-driven learning models have been described as associative, they are in fact discriminative (Ng & Jordan, 2002; Ramscar et al., 2010). In the light of this, it is worth considering the kind of cue and outcome representations that best support discriminative learning. Therefore in this section, we will describe some guidelines and heuristics for defining cue and outcome representations that can trigger discriminative learning dynamics. Generally the aim of a discriminative learning model is to discover a weighted combination of cues which best predicts the present outcome, or in other words, to discriminate the best cue structure given the present outcomes (Ramscar et al., 2010). Although the cue and outcome representations that are given to a model should be informed by theory, in order that the model can also contribute to theory, it can be useful to give it some leeway to discover a realistic structure in the cue weights within these representations. In contrast, if the scope of a defined cue structure in a model is too limited, the model will probably be less able to generate novel predictions and robust generalizations.

In what follows, we illustrate three important points that help improve cue representations with the aim of allowing a two-layer error-driven model to unfold its full discriminative mechanism, using Ramscar et al.,’s (2013) model of English plural acquisition. The task of this model was to predict the plural form, for example “mice”, of a noun based on cues from the environment, such as the items referred to by a specific noun, for example the presence of multiple mouse items. First, we conclude from the discussion about which levels of abstraction should be considered for the specification of cue representations, that including representations on multiple levels of abstraction can considerably increase the possibilities of a model to find predictive structure in cue representations given a set of outcome representations. Crucially, in addition, the discriminative view suggests that discrimination proceeds from high to low levels of abstraction, which can also be interpreted as coarse and detailed levels of discrimination.

Ramscar et al.,’s (2013) model of English plural acquisition is directly based on this idea. It includes representations of cues from the environment on multiple levels of abstraction in order to predict the plural form of an English noun (which can be regular or irregular): Ramscar et al., (2013) observed that early in learning, when the model has mainly seen regular plural forms (together with the objects appearing with them), the general cue *multiple items* is rated by the model to reliable predict the regular plural ending “-s”, leading to quick overregularization of the less frequently occurring nouns with irregular plural formation; only later, the model discovers more reliable, lower-level cues, such as *multiple mouse items* to predict specific plural forms (i.e., “mice”). Thus, in their case, the inclusion of representations on multiple levels of abstraction leads to the finding that during the process of discrimination, effects such as overregularization can occur.

Another example of the importance of considering levels of abstraction of cue representations in two-layer error-driven learning models comes from a recent proposal by Baayen and Hendrix (2017) who present a series of models that challenge Minsky and Papert’s (1969) historical claim that two-layer perceptrons (equivalent to the framework presented here) can solve only linearly separable problems. Contrary to this, Baayen and Hendrix (2017) show that when representations of an abstract nonlinear problem are on beforehand transformed into a linear domain, they can be solved by a two-layer error-driven learning network. Hence, while two-layer error-driven models cannot construct abstractions from the cue representations that they have access to, problems requiring such abstractions can still be investigated with such models, given that abstract cue representations are explicitly given to a model.

A second point, directly related to the different levels of abstraction of cue representations, is that only a cue structure where sets of cues overlap between different outcomes can trigger the full error-driven learning mechanism with cue and outcome competition. Notably, overlap can often be added to a set of cue representations by including representations on higher and lower levels of abstraction than seem intuitively necessary to predict different outcomes reliably (e.g., see: Baayen et al., 2016b; Hoppe et al., 2020; Ramscar et al., 2010). On the one hand, creating overlap with cue representations on higher levels of abstraction can be important for simulating the process of discrimination in an error-driven learning model: for example, in Ramscar et al.,’s (2013) model, the overregularization effect arises because, initially, more general and less informative cue representations are poorly discriminated from more detailed and more informative cue representations. On the other hand, overlapping low-level cues (e.g., the cue representations {*small*, *hopping*} in addition to {*dog*}) can be particularly useful when investigating how abstract representations might be learned (e.g., Baayen et al., 2016b; Hoppe et al., 2020; Ramscar et al., 2010).

A further important thing to note is that all representations in two-layer networks should intersect (i.e., no two sets of cues should be disjoint). The simplest way of ensuring this is to include a cue that occurs in all learning contexts (sometimes referred to as a context, environmental, or a *constant cue*^5^). Conceptually this cue can be thought of as corresponding to the properties of the environment that are constant, such as for example the presence of the learner, or that are undiscriminated, in Ramscar et al.’s (2013) model, for instance, the cue *stuff* was added to every cue set. Critically, from the perspective of the actual learning mechanism, the addition of this cue ensures that no sets of cues can be disjoint and that the amounts of error produced in different learning events always add up to zero. As a result, the cue weights learned will be in proportion to the whole learning system — basically this cue plays the same role as the intercept term in a regression model. Moreover, the inclusion of a representation for generally undiscriminated properties of the environment makes a model more realistic given the assumption that learning proceeds from perceiving the world as an undiscriminated set of items and events to learning to perceive predictive structures in the environment.

Third, Ramscar, Dye, and McCauley’s (2013) example illustrates how important it can be to consider the larger context of a problem, such as for example multiple modalities. While at a first glance, noun inflection might seem to be a problem that could be modeled using only phonological or morphological units (such as e.g., MacWhinney & Leinbach, 1991; Rumelhart & McClelland, 1986), the involvement of cues from the physical environment (i.e., semantics) have been shown to be essential to solving the problem (Ramscar, 2002). In addition, the larger context can also literally be just a larger system of cues and outcomes, which does not only involve the specific entities involved in the problem. Ramscar, Hendrix, Shaoul, Milin, & Baayen (2014), for example, investigate age effects in paired-associate learning of a small subset of noun associations by looking at how associations between words in the whole language develop over time for a single speaker. In this way, they show that the associations which a noun has built over a lifetime of language learning, directly influence how easily it can be associated with the nouns used in the paired-associate learning test, some of which are highly unlikely to appear in vicinity to the target noun in natural language.

To summarize then, these three factors, representations on multiple levels of abstraction, overlapping sets of cues, and inclusion of the larger context can make cue representations more discrimination-friendly in the sense that they define a problem which actually requires a discriminative learning mechanism. Crucially, even with a large number of cues, the model dynamics usually still stay accessible as only a small amount of the learned connection weights will be significantly different from zero (Arnold et al., 2017). In the light of these considerations, we would suggest that representations should err in the direction of richness rather than sparseness. The idea being that a model provided with sufficient “data” will probably learn to mirror predictive relationships between real items or events more closely and robustly than a model provided with a more limited context.

### Outcome representations

Thus far, we have mostly focused on cue representations and disregarded the impact of outcome representations, which are rarely discussed in the literature. Regarding discrimination they also have a less prominent role, as they are not the object of the discrimination process, such as cue representations, but the subject. Hence, usually outcome representations define the task at hand, for example, naming an object (where outcome representations would be word forms) as opposed to inferring what a speaker was communicating with a specific word (where outcome representations would be items or events from the environment or semantic representations). Furthermore, the level of abstraction of outcomes has a direct impact on the learned cue structure, as usually discrimination on different levels of abstraction stands in a trade-off: either focus has to be put on features which discriminate categories from each other, thus that are shared between items within a category, for example, features that discriminate dogs from rabbits, or focus has to be put on features which differ between items to discriminate them, for example, unique features of different dog breeds (Dye & Ramscar, 2009; Hoppe et al., 2020; Ramscar, 2013). Overall, the outcome structure determines what, that is, which cue structure, a model is going to learn. This also means that a two-layer error-driven learning model can only capture one learning process with one objective and cannot be compared with a process model or a cognitive architecture (e.g., ACT-R, Anderson 1996, 2005).

Finally, it’s important to stress that the cue and outcome representations employed are a defining part of a model (Bröker & Ramscar, 2020) which can set apart different kinds of models based on the same error-driven learning mechanism and on the same network architecture (Note that we use the term *model* here in its more specific sense as consisting not only of a learning mechanism and a network architecture, but also of specific cue and outcome representations). Because of this, and because cue and outcome representations inevitably embody the theoretical assumptions of modelers it seems fair to say that the various error-driven learning models in cognitive science do not form a coherent theoretical class of models, a point illustrated for example by the vast differences between error-driven grammar learning models (MacWhinney & Leinbach, 1991; Ramscar et al., 2013; Rumelhart & McClelland, 1986), which vary widely in their data structure and theoretical assumptions and in to what extent they treat their tasks as discrimination problems.

## Discussion

Historically, not long after two-layer error-driven learning networks were initially introduced into the cognitive sciences, the focus of the field shifted to multi-layer networks. One reason for this was that it was thought that only they could resolve the limitations of two-layer networks that have been highlighted at the time (Minsky & Papert, 1969). One consequence of this was that the degree to which two-layer networks might still contribute to our understanding of cognition was largely left unexplored over the following decades (a factor that was further compounded by the limited available computational resources at the time). It is only recently that researchers have begun to reexamine the degree to which two-layer networks might still contribute to theoretical understanding. On the one hand, new evidence suggests that some of the originally claimed limitations can be overcome, or rather that they do not need to be overcome if we, for example, can assume different stimulus representations (e.g., Baayen & Hendrix, 2017). On the other hand, some aspects of simple error-driven learning models that have been claimed to be limitations can in fact be interpreted as advantages when it comes to using these models to understand the concrete mechanisms underlying human or animal behavior.

In this regard it is important to note that the main point of exploring a learning phenomenon with a simple two-layer network is to maximize its interpretability. This means that the problem must be abstracted to a degree which, first, makes the workings of the model directly accessible, and second, makes it possible to relate them directly to the basic error-driven learning mechanism. Two-layer models achieve this goal by their very simplicity, which minimizes free parameters and in this way makes transparent the basic error-driven learning mechanism. This simplicity helps to ensure that a model’s behavior can be directly attributed to the main error-driven learning mechanisms (association and dissociation) and the resulting dynamics (cue and outcome competition). Consider for example different approaches to modelling the process of forgetting: on the one hand in error-driven learning, forgetting is simulated via interference dynamics, which directly result from the process of dissociation; on the other hand, there is evidence (Ramscar et al., 2017) that this produces behavior very similar to behavior produced by time decay dynamics, which are usually implemented as a separate process in learning models based solely on association (Anderson, 1996; Pavlik & Anderson, 2005). The question whether or to what extent interference or time decay can account for forgetting is still discussed in the memory literature (e.g., Hardt, Nader, & Nadel, 2013; Oberauer & Lewandowsky, 2008). However, comparing the two models shows how in the interference model, forgetting arises out of the basic model dynamics, while in the time decay model, the mechanism of forgetting needs to assume an additional process. While (and maybe also because of the fact that) it is rather difficult to separate the passing of time and interference, the interference explanation seems to be more parsimonious since it suggests that forgetting is actually inherent to the learning mechanism itself.

It is clear that the increased interpretability of these models comes at the cost of scope, and historically, many problems that researchers have tried to tackle might simply not be suited to be modeled this way. If we, for example, look at the long list of “failures” brought forward by Miller et al., (1995), while a few of them might be solved by reframing the learning problem or input and output representations, for most of these problems it is likely that they are simply not really suitable for modeling in a two-layer error-driven learning network. One solution, that has been often applied but comes with the problem of not contributing much theoretical insights (see also Section “?? ??”), is to keep the learning mechanism and add additional parameters that can make the mechanism work for problems, such as for example overshadowing (as it is, e.g., done by Rescorla and Wagner, 1972). Another solution is to acknowledge the limitations of these simple models and to draw on different, or additional mechanisms or more complex network architectures. Regarding the case of overshadowing, Rokers et al., (2002) provide an example of how a combination of two multi-layer error-driven networks connected by a feedback mechanism can simulate how more or less salient cues act differently in a blocking paradigm tested on rats. Their model is directly informed by research on cortico-hippocampal networks and implements a postulated mechanism in the septal region that modulates input representations during learning. Importantly, this highlights another limitation of simple two-layer networks: although they can discriminate between input representations (potentially at different levels of abstraction) and in this sense *learn* which representations they need to predict the outcomes defining a given task, they cannot *learn* (in the sense of *form*) these representations during the same learning process with the same outcomes (Ramscar, 2021). In order for the latter process to occur, the more abstract cue representations that need to be learned would somehow need to be given to the model as outcomes (besides the representations defining the task at hand) *and* as cues. It is far from clear how this can be done in a two-layer network, yet it is exactly what happens in a multi-layer network, where hidden units can serve both as outcomes to input representations and cues to output representations (or further hidden units).

This leads directly to another point which is important for understanding the scope of these models: they do not (or should not) aspire to be complete process models. Although error-driven learning was first introduced into psychology in what were essentially blackbox empiricist terms (i.e., as models of “the learning process"), our increasing understanding of the neurobiology of learning and the inherent complexity of human neurocognitive architecture makes clear that no single error-driven learning network, regardless of its complexity, can be realistically considered to be a ‘complete’ model of how the cognitive system learns. However, in contrast to more holistic symbolic cognitive architectures, simple error-driven learning models have nevertheless turned out to be particularly helpful regarding the investigation of the neural underpinnings of more complex cognitive processes: the fact that they are able to capture in some detail the specific aspects of learning given a specific situation and a specific set of assumptions has enabled researchers to employ these models to predict the behavior of specific neural mechanisms in learning (Schultz, 2000; Schultz, Dayan, & Montague, 1997; Waelti, Dickinson, & Schultz, 2001). This work has allowed to relate the behavior of different brain regions in learning to the different subprocesses of more complex network architectures (Mack, Love, & Preston, 2016; Mack, Love, & Preston, 2018; Mack, Preston, & Love, 2020), as well as to connect observed changes in learning in childhood to the development of a more complex network learning architecture in development (Ramscar & Gitcho, 2007; Thompson-Schill, Ramscar, & Chrysikou, 2009).

These findings further support the idea that while simple error-driven learning networks are ultimately limited in their computational power as compared to more complex modeling approaches, it remains to be seen whether the specific characteristics causing this reduction in power are in fact limitations. One such arguable limitation is, for example, that error-driven learning based on the delta rule, as we present it here, is a discrete process, and thus cannot directly process continuous input. However, it can still be the case that — given that from a scientific perspective, simulations are ultimately aimed at increasing understanding — this simplification can nevertheless illuminate mechanisms of learning from continuous input. In fact, whether mechanisms in cognitive processing and learning are discrete or continuous is often a question under discussion and, interestingly in many cases, such mechanisms can be modeled by both discrete and continuous models (e.g., Miller, 1988; van Der Wel, Eder, Mitchel, Walsh, & Rosenbaum, 2009). If we assume that discretization is a process of abstraction from what are originally continuous sensory signals, this then raises the question what kind of levels of abstraction are necessary to model a problem, which can be investigated very well with two-layer error-driven learning models. First, because of the fact that in modeling with two-layer networks, representations on different levels of abstraction have to be chosen explicitly, this question is often naturally considered during the modeling process. Second, as computational resources have grown, it has become possible to approximate a continuous signal surprisingly closely with a two-layer error-driven model (i.e, a continuous acoustic speech signal: Arnold et al., 2017), hence enlarging the range of possible levels of abstract representations to explore. Two recent studies investigating the required levels of abstraction in language processing with two-layer error-driven learning models find that surprisingly low levels of abstraction are required for word processing: Baayen et al., (2016b) find that a model with speech input which is not segmented into words but into a of triphones shows behavior which has been before explained by segmentation; Arnold et al., (2017) find that a model trained on a close approximation of a continuous speech signal shows similar performance in word recognition to human participants. In summary then, it seems that although simple two-layer error-driven models cannot model continuous processing directly, they can be used to explore questions in relation to the kind of representations and levels of abstraction that are theoretically needed to solve problems of cognitive processing or learning even when it comes to continuous processes.

Another important characteristic of simple two-layer error-driven learning models is that they by definition are unable to transform their input representations during learning (beyond simple weight adjustments). The advantages that multi-layer networks bring in this regard is one of the main reasons for their success in many engineering applications. Crucially, multi-layer networks solve two problems that emerge in two-layer networks: the problem that researchers are constrained by their theory and underlying assumptions in choosing a set of input representations and the problem that any attempt of hand coding a complete set of possible representations leads to a combinatorial explosion whenever cue representations on multiple levels of abstraction are required. However, at the same time and somewhat paradoxically, this weakness may actually be a strength when it comes to using simple networks as theoretical tools. On the one hand, the fact that two-layer models cannot learn internal representations, clearly limits their capacity to solve a complex task by considering only simple input representations especially when the abstract representations actually required to solve the task are unknown. On the other hand, because in simple two-layer networks all cue and outcome representations have to be chosen explicitly, researchers are forced to attend more closely to the cue and outcome representations selected, which in turn facilitates the generation of concrete theoretical insights in this regard. In particular, the very simplicity of these models can serve to make explicit the consequences of representational assumptions — for example, whether a stimulus is likely to be perceived as a chunk or as a set of elements, or the degree to which two given outcomes are in fact discriminable to a learner — that can be obscured by more powerful models. In contrast, because representations learned internally in complex hidden layers are not directly accessible to researchers, the use of multi-layer networks can lead to situations where researchers are able to simulate hypothesized behavior in a given task without fully understanding exactly why a given simulation actually works. Hence, while multi-layer networks can simulate the construction of abstract representations, two-layer networks use explicitly chosen representations to investigate how specific representations are related to a learned behavior. Consequently, both approaches contribute to the understanding of the learning process on different levels of abstraction and therefore, in the best case, they should inform research in a complementary way. For current purposes, the combination of these approaches also helps clarify how in fact the learning of predictive input representations in an abstract non-linear space is ultimately the key mechanism of error-driven learning: first, this is exactly what happens in the hidden layers of deep neural networks and, second, we have seen that already to model simple behavioral results, such abstract and non-linear representations can also be helpful in a simple two-layer model.

These last considerations show again the trade-off between detail and abstraction in modeling. As Bonini’s paradox suggests, increasing complexity often comes at the cost of understanding. Notwithstanding the fact that approaches of different degrees of complexity and abstraction are necessary to resolve this modeling *paradox*, two-layer error-driven networks can, when sensitively and appropriately employed, serve as an opposing force to the tendency towards increasingly complex models. By placing the focus on the learning process itself, this simple framework is a valuable tool for the study of error-driven learning, which is not only an ubiquitous mechanism in today’s models of learning but also clearly has much to contribute when it comes to the development of theories of cognition. In this way, a careful analysis of the basic error-driven learning mechanism leads to the conclusion that this learning mechanism is inherently discriminative which in turn implies that it might be most productively applied within a discriminative theory of learning and processing.

## Appendix: : Adjusted EDL weight updates for illustration purposes

To illustrate the contributions of both cue and outcome competition (see Fig. 5), we created two adjusted versions of the weight update, one with disabled cue competition and one with disabled outcome competition.

To disable cue competition, errors are not calculated relative to the outcome activation, but only relative to the weight of the current cue to the current outcome:

6 $$  {{\varDelta}} V_{ij}^{t} = \left\{\begin{array}{lll} 0   & \text{, cue } i \text{ absent} \\ \eta(1 - v_{ij}^{t})   & \text{, cue } i \text{ and outcome } j \text{ present } \\ \eta(0 - v_{ij}^{t}) & \text{, cue } i \text{ present but outcome} j \text{ absent} \end{array}\right. $$

To disable outcome competition, the third case of the weight update is ignored during learning. As a result, only weights to present outcome are updated:

7 $$  {{\varDelta}} V_{ij}^{t} = \left\{\begin{array}{lll} 0   & \text{, cue } i \text{ absent} \\ \eta(1 - ac{t_{j}^{t}})   & \text{, cue } i \text{ and outcome } j \text{ present} \\ 0 & \text{, cue } i \text{ present but outcome } j \text{ absent} \end{array}\right. $$

## Footnotes

1 This kind of network architecture is often also referred to as a single-layer network, following a convention of not counting the layer of input units in order to contrast multi-layer architectures which include at least one layer of hidden units. However, given that there is a layer of input and an output units, we use the term two-layer for this architecture

2 In terms of classical conditioning, *conditioned stimulus (CS)* corresponds to *cue* and *unconditioned stimulus (US)* corresponds to *outcome*. Often, only two outcomes are considered: reinforcement (+) or non-reinforcement (-) of the US, leading to notations such as “CUE1+” and “CUE2-”.

3 Theoretically Rescorla and Wagner (1972) had assumed different learning rates for different outcomes but practically they presented only models considering two outcomes, reinforcement and non-reinforcement.

4 This illustrates that error-driven learning does *not* assume that weights between cues and outcomes decay over time.

5 Note that in our example simulations we deliberately did not include a constant cue in order to make them easier to understand, also when they included disjoint cue sets, such as the examples in Figs. 6, 7b, and 8b. Crucially, however, activations in these cases would not be affected by adding a constant cue to each cue set, given that the constant cue is also included in the calculation of the activations.
